# Optical quantum technologies with hexagonal boron nitride single photon sources

**DOI:** 10.1038/s41598-021-90804-4

**Published:** 2021-06-10

**Authors:** Akbar Basha Dhu-al-jalali-wal-ikram Shaik, Penchalaiah Palla

**Affiliations:** Center for Nanotechnology Research & Department of Micro and Nanoelectronics, School of Electronics Engineering, Vellore Institute of Technology (VIT), Vellore, Tamil Nadu 632014 India

**Keywords:** Nanoscience and technology, Single photons and quantum effects

## Abstract

Single photon quantum emitters are important building blocks of optical quantum technologies. Hexagonal boron nitride (hBN), an atomically thin wide band gap two dimensional material, hosts robust, optically active luminescent point defects, which are known to reduce phonon lifetimes, promises as a stable single-photon source at room temperature. In this Review, we present the recent advances in hBN quantum light emission, comparisons with other 2D material based quantum sources and analyze the performance of hBN quantum emitters. We also discuss state-of-the-art stable single photon emitter’s fabrication in UV, visible and near IR regions, their activation, characterization techniques, photostability towards a wide range of operating temperatures and harsh environments, Density-functional theory predictions of possible hBN defect structures for single photon emission in UV to IR regions and applications of single photon sources in quantum communication and quantum photonic circuits with associated potential obstacles.

## Introduction

Quantum information science (also known as quantum information studies) is an emerging interdisciplinary research field of science and technology, which is primarily concerned with analysis, processing, storage, retrieval and secure transmission of information through the set of quantum mechanical principles. The information in quantum science is inscribed using various physical properties of quantum elementary particles such as spin of an electron (spintronics) and polarization of a single photon (photonics) etc.

Integrated quantum photonics is a sub-field of QIS (quantum information science), which uses photonic integrated circuits to architect quantum states of photons for intersecting the quantum applications.

### What is a single photon emitter?

In a broad perspective, a single photon is attained by two main mechanisms such as excitation and followed by spontaneous emission. The elementary particle, electron either in a molecule or in atom always tends to exist in ground state (un-excited state).

**Figure 1:**
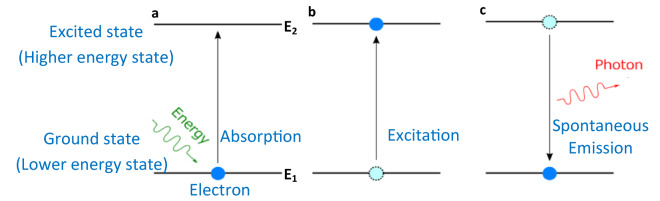
Pictorial representation of absorption, excitation and spontaneous emission. (**a**, **b**) electron in ground state absorbing energy E = E_2_ − E_1_ and moved to excited state. (**c**) After few nanoseconds the electron decays back to ground state and releases the absorbed energy in the form of a photon. The spontaneous emission due to optical excitation is called photoluminescence and the spontaneous emission due to electrical excitation is called electroluminescence.

When this electron in ground state is excited (absorbs energy), moves to higher energy state (excited state) as shown in Fig. 1a, b. This energy absorption can be accomplished either by photoexcitation (ground state electron absorbs a photon of light and gains its energy) or electrical excitation (ground state electron absorbs energy from another excited electron).

This excited electron stays not more than few nanoseconds in higher energy state (excited state) and spontaneously return back to lower energy state (ground state). During this decay, the electron emits the absorbed energy in the form of radiation as shown in Fig. 1c.

This subtler process is called spontaneous emission which leads to emit a single photon and the quantum level light sources which emits a single photon (elementary particle of light) per one excitation cycle (combination of one excitation and spontaneous emission) is called single photon emitters often called as quantum emitters.

### Why single photon emitter?

The numerous scientists and engineers working in the domain of quantum photonics and Integrated quantum photonics, worldwide predicted and proved that these quantum technologies would overcome the limitations^1,2^ of classical computing and data communications technologies. Quantum computers enhance the processing speed and efficiency than classical computing using qubits^3^. For quantum information processing, in optical systems, the unit of light ‘photon’ is used to represent a qubit.

Secure information transfer by quantum communication (in particular, quantum cryptography technique) is highly in demand for defense organizations and financial institutions^4^. These computing and communication techniques employs polarize states of single photons to encode the information.

### State-of-the-art single photon emitters in 2D materials

However, traditional single photon emitters in three dimensional materials limits the photon extraction efficiency and integration ability to photonic circuits due to high refractive index.

The discovery of 2D materials from insulating hBN (hexagonal boron nitride), semiconducting TMDs (transition metal dichalcogenides) to semi metallic graphene exhibits exceptional properties such as Quantum confinement in the direction perpendicular to 2D plane leads to novel electronic and optical properties. These novel properties significantly different from their bulk counterpart^5–8^. These naturally passivated 2D materials, without any dangling bonds, makes it easier to integrate with photonic structures such as waveguides^9–12^ and cavities^13–17^. Another notable property of these materials is that these materials strongly interacts with light^18^.

The single photon emission in TMDs was initiated by exciton formation (bound state of a free electron and an electron–hole, attracted to each other by electrostatic force) generated by either optical or electrical excitation and a single photon is emitted during recombination of this electron hole pair.

Generally the excitons formed in TMDs are of wannier–mott kind, which tend to diffuse (not localized) across lattice. The point defects and crystal imperfections in the TMDs (WSe_2_, WS_2_, MoSe_2_, and Mos_2_) and GaSe^19–46^, found to trap and localize the excitons at particular location, which in turn leads to localized single photon emission at cryogenic temperatures.

Particularly, in case of WSe_2,_ intentionally induced strain gradients (by nanopillars) are used to funnel the excitons for effective single photon emission^47,48^. Electrostatic potentials in moiré patterns leads to effective exciton trapping, which in turn leads to single photon emission in bilayer TMDs, as observed in ref^49–53^.

On the other hand, in wideband semiconductors like hBN, the point defects acts as flawless single photon emitters. These point defects tend to exhibit intermediate energy states (un-occupied and electron occupied energy levels) in between the conduction band edge and valence band edge of the energy bandgap of the material and the electrons in occupied energy levels found to exist in paramagnetic form. Due to optical excitation, these paramagnetic electrons excites to higher un-occupied energy states and results in spontaneous single photon emission at wide range of temperatures^54,55^, which founds to be more suitable for quantum applications.

Particularly, in hBN, enhancement of single photon emission was studied via external electric fields in ref^56^ and via external magnetic fields in ref^57^. Tunable quantum emission from very recent 2D heterogeneous structures such as graphene/hBN/Wx_2_ or Mox_2_/hBN/graphene (where x = Se or S) opens the door for fascinating applications like quantum memory^58^, quantum imaging and metrology^59–61^.

Achieving bright and stable single photon emitters in a single chip, which covers the emission range from UV to IR spectrum and works over a wide range of temperatures is really significant because each range of SPS (single photon source) have their own on-demand unique applications.

**Figure 2:**
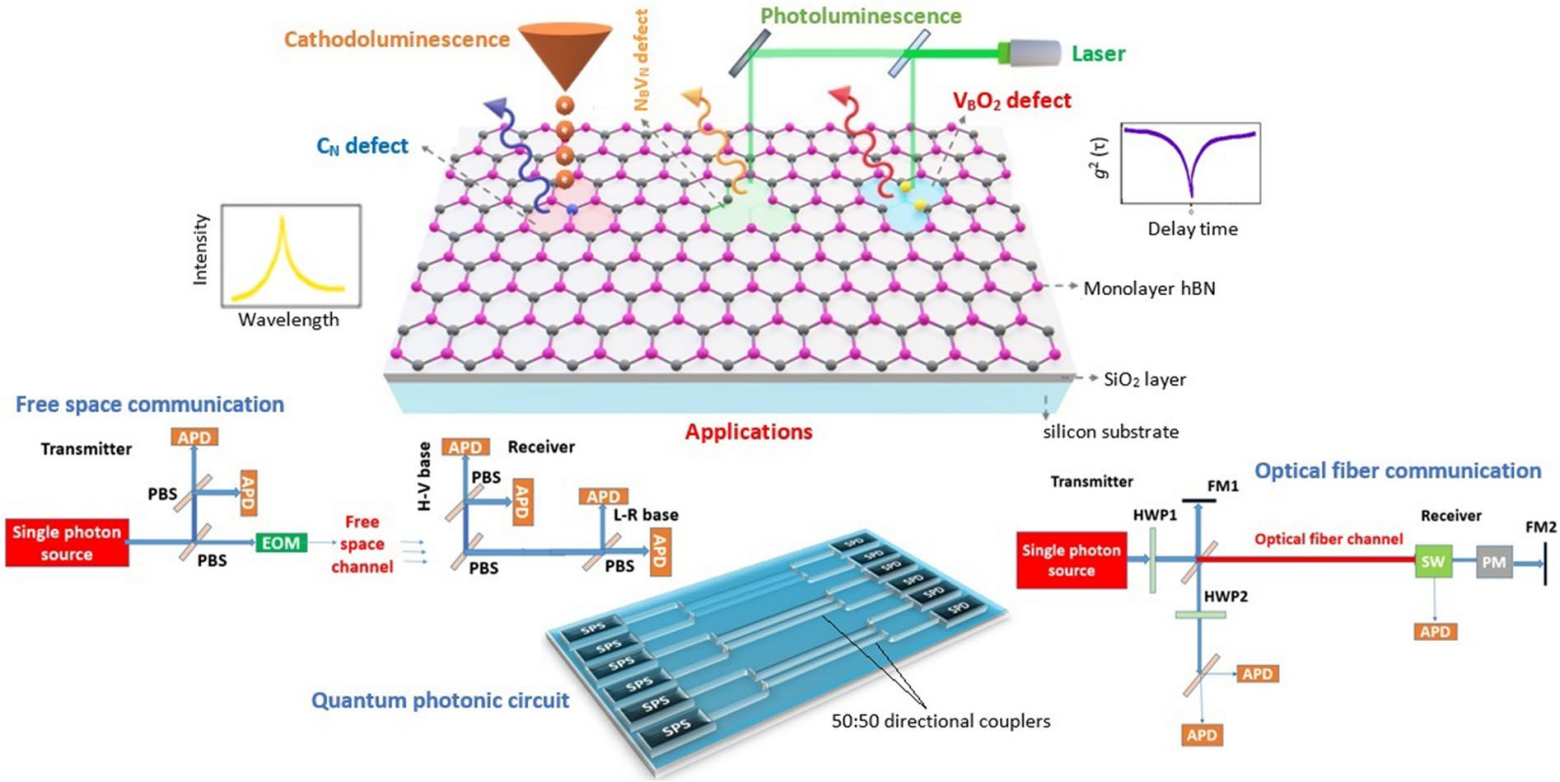
Schematic representation of three main quantum emitters hosts in hBN on Si/SiO_2_ substrate, emitting single photons of different energies and their unique applications. hBN monolayer by alternating boron (pink) and nitrogen (grey) atoms, on Si/SiO_2_ substrate. Three main quantum emitters (among various luminescent point defects predicted) namely C_N_ defect (carbon (blue) replaces nitrogen atom) emits single photon in UV region, finds an application in free space quantum communication^67^. N_B_V_N_ defect (nitrogen vacancy and boron replaces nitrogen) emits single photon in visible region, finds an application in quantum photonic circuits for quantum computing^68^ and V_B_O_2_ defect (boron vacancy with oxygen (yellow) atoms) emits single photon in near IR region, suitable for optical fiber quantum communication^69^.

Among various luminescent point defects predicted (more details given in Fig. 11)^62^, the schematic representation of three main (C_N_, N_B_V_N_ and V_B_O_2_) point defects, hosts in hBN are shown in Fig. 2. These point defects covers the emission range from UV to near IR region as listed in Table 1 and these also consistent with experimental studies performed as given in references^63–66^.

**Table 1:** Emission wavelength range of hBN quantum emitters.

Predicted defect structure	Emission wavelength range (energy range)	Remarks	References
S-based defects	~ 639 nm to 984 nm (1.26 to 1.94 ) eV	The emission wavelength range of hBN quantum emitters covers the electromagnetic spectrum from UV to near IR region	^62^
Si-based defects	~ 1.99 mm to 325 nm (0.62 to 3.81) eV		
Stone–Wales defects	~ 459 nm (2.70) eV		
C-based defects	~ 1.11 mm to 310 nm (1.11 to 4) eV		
O-based defects	~ 650 nm to 700 nm (1.7 to 1.9) eV		
native defects	~ 300 nm to 620 nm (2 to 4) eV		
C_N_ defect	~ 302 nm (4.1) eV		^63^
N_B_V_N_ defect	576–652 nm (1.9 to 2.15) eV		^64,65^
V_B_O_2_ defect	~ 700 nm (1.77) eV		^66^

Among various luminescent point defects predicted in hBN flakes (as shown in Fig. 11), C_N,_ N_B_V_N_ and V_B_O_2_ defects are consistent with recent experimental studies performed. In N_B_V_N_ point defect, variation in emission energy is due to variations in local strain and dielectric environment.

This Review provides a snapshot of current research on single photon emitters hosts in hBN as well as other 2D materials, their functionalities, potential quantum applications and limitations to overcome.

## Salient features of hexagonal boron nitride and single photon emitter

### Hexagonal boron nitride

hBN is an isostructural material to graphite. Layered hBN also known as white graphene due to its highly transparent nature and the first known natural hyperbolic material, i.e. in-plane bonds are stronger than out-of-plane bonds.

**Figure 3:**
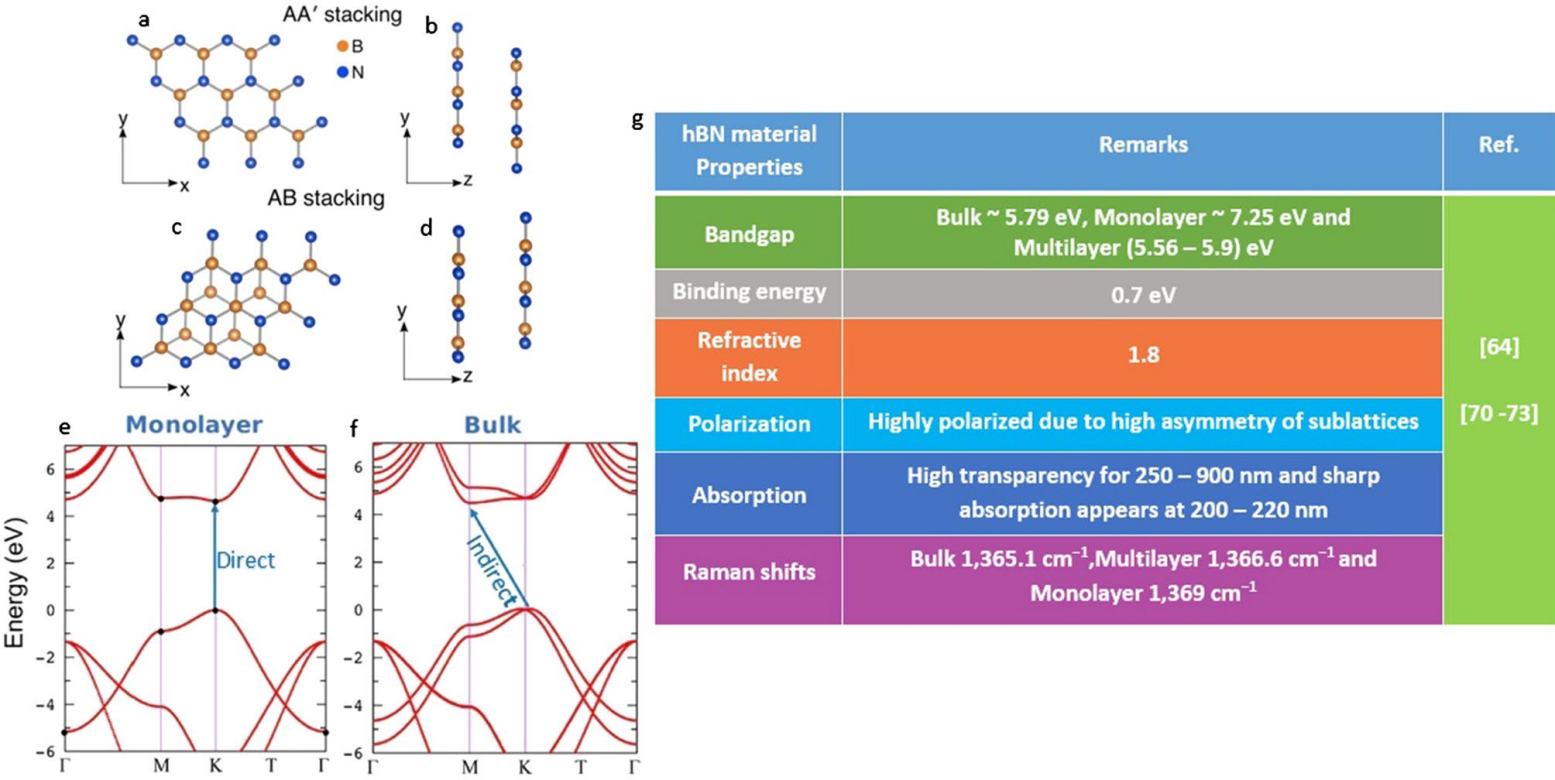
Schematic representation of hBN stacking^74^, electronic band structure of monolayer and Bulk hBN^75^ and electrical/optical/crystal properties of hBN material. (**a**, **b**) Top view and side view of AA’ stacking. (**c**, **d**) Top view and side view of AB stacking. (**e**, **f**) Electronic band structure of monolayer and bulk hBN with direct and indirect bandgaps respectively. (**g**) General properties of hBN material and similar Raman shifts (around values) can be observed for high quality crystals.

Alternating boron (B) and nitrogen (N) atoms in its crystal structure, monolayer hBN is a sp^2^ bonded layer material. In (bulk or multilayer) hBN, these layers are arranged either with AA' stacking (B atoms of one layer are positioned above the N atoms of another layer or vice versa for N atoms of one layer w.r.t B atoms of another layer) as shown in Fig. 3a, b or AB stacking (B and N atoms of one layer are translated w.r.t another layer) configuration as shown in Fig. 3c, d.

These individual layers are packed together by weak van der Waals forces. The inter layer interactions affects the band structure of material and the variation in electronic band structure of monolayer and bulk hBN is shown in Fig. 3e, f. The monolayer hBN is a direct bandgap semiconductor material with 7.25 eV at high symmetry K point. As the number of layers increases, it becomes optically inactive (bulk) semiconductor with indirect bandgap of 5.79 eV with conduction band minimum at M and valence band maximum at K points.

The variation in bandgap of hBN and their corresponding Raman shifts essentially depends on interlayer interactions and number of individual layers stacked. Owing to larger bandgap, hBN exhibits high transparency of electromagnetic spectrum. hBN also exhibits relatively high refractive index and other electrical/optical/crystal properties of hBN were listed in Fig. 3g.

### Single photon emitter

**Figure 4:**
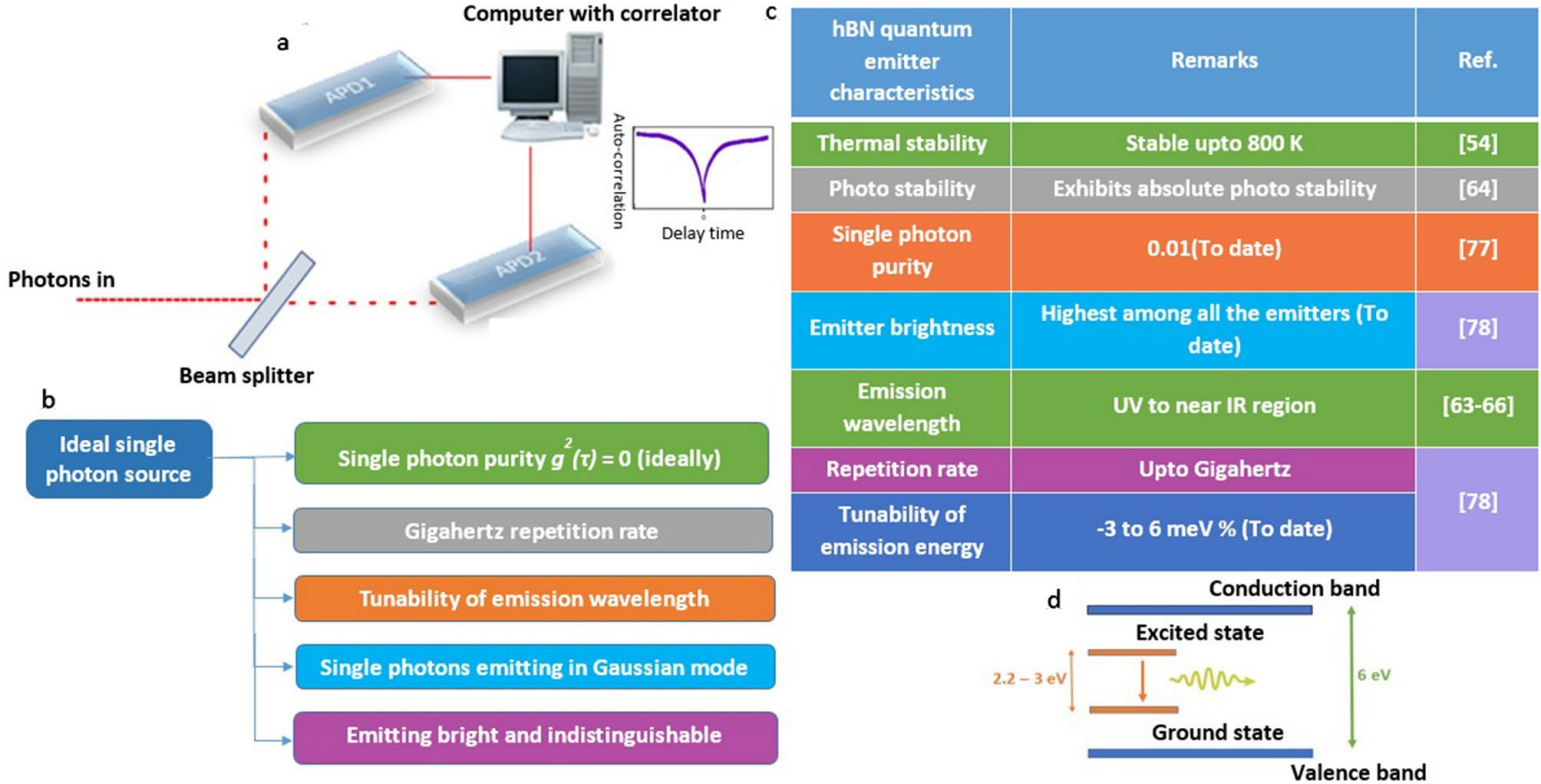
Schematic of HBT interferometer, important features of an ideal single photon source and experimentally observed quantum emitter characteristics, pictographic representation of atomic behaviour of defects within the host bandgap (**a**) Schematic representation of HBT interferometer working mechanism and resultant second order autocorrelation curve representing characteristics of a single photon emitter. (**b**) Important features of an ideal single photon source. (**c**) Experimentally observed some of the quantum emitter characteristics hosts in hBN, in which characteristic stability upto 800 K and single photon purity 0.01 makes a highest record among all the 2D materials (to date). (**d**) The energy band diagram of an hBN host with ~ 6 eV bandgap. A luminescent point defect in hBN (with energy range ~ 2.2–3 eV) exhibits an artificial atom kind of behaviour with ground and excited states.

The single photon emitter is a non-classical light source, emits only one photon per excitation cycle, observed experimentally using a Hanbury, Brown and Twiss (HBT) interferometer^76^ as shown in Fig. 4a. The elementary particle ‘photon’ cannot split further after passing through 50:50 beam splitter (as conventional light do), will be detected at any of the APD (avalanche photodiode) in HBT interferometer.

At zero delay time, the second order autocorrelation curve dips below 0.5 indicates the characteristics of a single photon emitter.

The single photon purity (represented by autocorrelation function g^2^ (τ)) is the main characteristic of a quantum emitter and other important features of an ideal single photon source (quantum emitter) to be considered while developing for quantum applications is listed in Fig. 4b and experimentally observed some of the quantum emitter characteristics of luminescent point defects in hBN are listed in Fig. 4c.

Luminescent point defects in hBN acts as excellent single photons emitters, can be described as an artificial atom having ground and excited states within the host bandgap as shown in Fig. 4d.

The nature of luminescent point defects can be precisely understood by study of additional photophysical characteristics as listed in Table 2 and corresponding values observed in hBN quantum emitters were listed in Table 3.

**Table 2:**
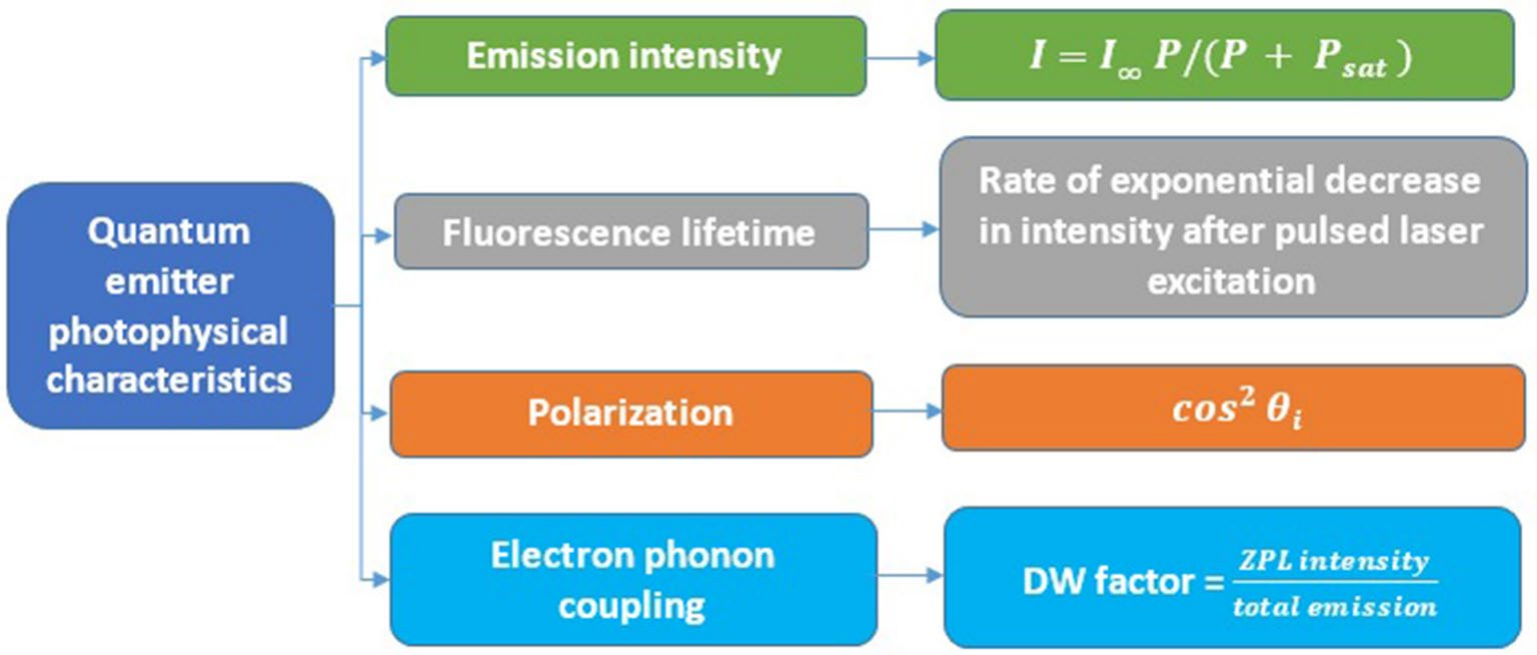
Photophysical characteristics of quantum emitters.

The photophysical characteristics of quantum emitters to characterize the nature of defect and their corresponding fitting equations where $I =$ Fluorescence intensity, $I_{\infty } =$ emission rate, $P = {\text{laser}}\;{\text{excitation}}\;{\text{power}}$, $P_{sat}$ = excitation power at saturation, $\theta_{i}$** = **angle between the initial polarization direction of light and transmission axis of the polarizer and DW = Debye–Waller.

**Table 3:** Photophysical characteristics values observed in hBN quantum emitters.

Photophysical characteristics of hBN quantum emitters	Remarks	References
Emission intensity	Highest reported (To date)	^64,77,78^
Fluorescence lifetime	1.53–2.88 ns	
Polarization	Linearly polarized dipole transition	
Electron phonon coupling (DW factor)	0.82	

All the experimental values were obtained at room temperature.

Polarization measurements for 103 isolated luminescent point defects in hBN (Zero Phonon Line (ZPL) range of ∼550–740 nm), revealed that the absorption and emission dipoles are frequently misaligned. This dipole misalignment is framed as:

$${\text{Dipole}}\;{\text{misalignment}}\;\Delta\uptheta = {\text{ZPL}}\;\left( {{\text{energy}}} \right){-}{\text{exciting}}\;{\text{light}}\;{\text{energy}}\;\left( {\Delta {\text{E}}} \right).$$

The possible range of dipole misalignments, responsible mechanisms and their influence on light absorption are listed in Table 4.

**Table 4:** Range of dipole misalignments, responsible mechanisms and their influence on light absorption.

Range of dipole misalignment (Δθ)	Responsible mechanism	Influence on light absorption	References
Δθ ≈ 0°	If excitation light energy (ΔE) coincides with an allowed phonon energy in hBN	Direct absorption of defect	^79,80^
0° ≤ Δθ ≤ 90°	If excitation light energy (ΔE) exceeds the maximum phonon energy in hBN	Indirect absorption through a third intermediate electronic state	

No dipole misalignments is observed when excitation light energy coincides with allowed phonon energy in hBN, results in direct absorption of defect. Dipole misalignment value ranges between 0° and 90° if excitation light energy exceeds maximum phonon energy in hBN and indirect absorption of defect takes place.

## Contrasting hBN quantum emission characteristics with other 2D materials and encapsulation of existing and emerging single photon sources

The comparison of some of the quantum emitter characteristics among hBN and 2D TMDs were listed in Table 5. Among all the atomically thin materials, hBN found to one step ahead in furnishing supreme quantum emitters close to ideal characteristics.

**Table 5:** Comparison of some of the single photon emitter characteristics between hBN and other 2D TMDs.

2D materials	Emission wavelength	Single photon purity *g*^*2*^(0)	Life time	(ZPL) line width	Operation temperature	References
hBN	UV–near IR	0.01	1.53–2.88 ns	∼ 0.21 μeV	Up to 800 K	^19–46^ ^54^ ^63–66^ ^77^ ^78^
MoS_2_	∼ 550 nm	–	< 150 ps	0.5–6 meV	Cryogenic temperatures	
WSe_2_	∼ 730–750 nm	0.02	100 ps , 2–225 ns	10 μeV		
WS_2_	∼ 640 nm	0.31	1.4 ns	3 meV		
MoSe_2_	∼ 770 nm	–	–	150–500 μeV		
GaSe	∼ 600 nm	0.33	5–22 ns	3.7 meV, 5.2 meV		

Observed variation in single photon emitter characteristics between hBN and other 2D TMDs in which hBN is found to be unique to operate up to 800 K and high single photon purity.

The advantages and drawbacks of existing, emerging single photon sources from single atom to very recent TMDs and 2D heterostructures are represented in Table 6.

**Table 6:** Comparison of existing and emerging single photon sources.

Technology	Source type	Emission wavelength	Advantages	Drawbacks	References
Existing sources	Single atom	Atomic line spectrum (Rubidium atom)	Better single photon purity	Operation at cryogenic temperatures and multiple atom effects	^81,82^
	Single ion	Atomic line spectrum (calcium ion)	Possibility of trapping single ion inside optical cavity	Reduction of single photon emission at the end of excitation pulse and Operation at cryogenic temperatures	^83,84^
	Single molecule	500–750 nm (terrylene molecule)	Operation at RT	Poor single photon purity and indistinguishability between photons of same molecule	^85,86^
Emerging sources	Color centers in diamond	640–800 nm	Photostable and operation at RT	Color centers are not identical and emitter brightness reduces at higher temperatures	^87–91^
	Quantum dots	510–690 nm (InP/ CdSe) and 700–1000 nm (InAs/AlGaAs)	Better integration with quantum photonic circuits and devices	Operation at cryogenic temperatures	^92–94^
	TMDs	~ 640 nm (WS_2_), ~ 600 nm (GaSe), ~ 770 nm (MoSe_2_), ~ 730–750 nm (WSe_2_)			^19–46^
	Carbon nanotubes (0.78 nm)	1,100–1,300 nm		Low brightness and single photon purity	^95^
Recent emerging sources	2D heterostructures	~ 700–800 nm	Enhances the quantum photonic applications	Operation at cryogenic temperatures	^49–53,58–61^

Overall comparison of emission wavelength, advantages and drawbacks of existing and emerging single photon sources from single atom to very recent TMDs and 2D heterostructures.

## Fabrication and activation processes of quantum emitters in hBN

### Bulk hBN

In bulk hBN^96^, emitters are found to be activated by annealing the samples for 30 min at 850 °C under 0.5 or 1 torr of argon. The argon gas is selected to prevent oxidation. Annealing at different temperatures found that fluorescence intensity is maximum at 850 °C.

To date, no superior techniques were experimented to fabricate the new emitters in bulk hBN, as the emitters in bulk hBN faces the disadvantages as listed in Table 10.

### CVD grown and exfoliated multi, single hBN layers

**Figure 5:**
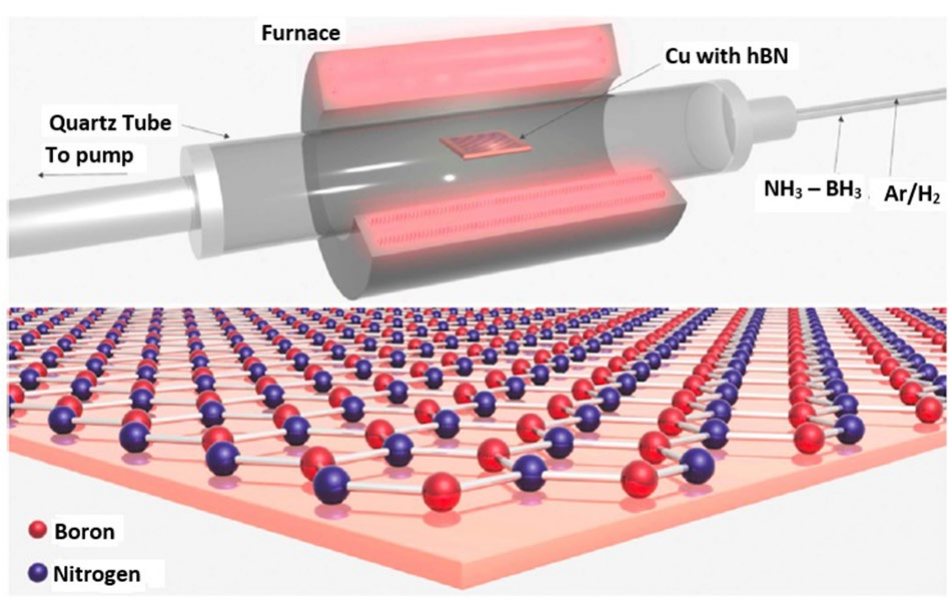
Schematic representation of a LPCVD grown hBN multilayer in quartz tube^97^. hBN multilayer growth on Cu foil in a quartz tube furnace at 1030 °C Temp. Ammonia borane (NH_3_-BH_3_) as a precursor and Ar/H_2_ as carrier gas.

hBN multilayers grown using low-pressure chemical vapor deposition (LPCVD) technique^97^, found to host stable quantum emitters around 200 (per 10 × 10 µm^2^ area) at room temperature. The growth technique was performed in quartz tube as schematic shown in Fig. 5. This LPCVD grown hBN multilayers are usually 1.4 nm (3 to 4 monolayers) thickness.

More than 85% of emitters exhibit ZPL around 580 nm, with single photon purity $g^{2} \left( 0 \right)$ less than 0.5 and absolute photostability. No post growth annealing was performed for emitter activation or stabilization which is contrast to the emitter fabrication techniques observed in exfoliated multilayer hBN flakes^98^.

**Figure 6:**
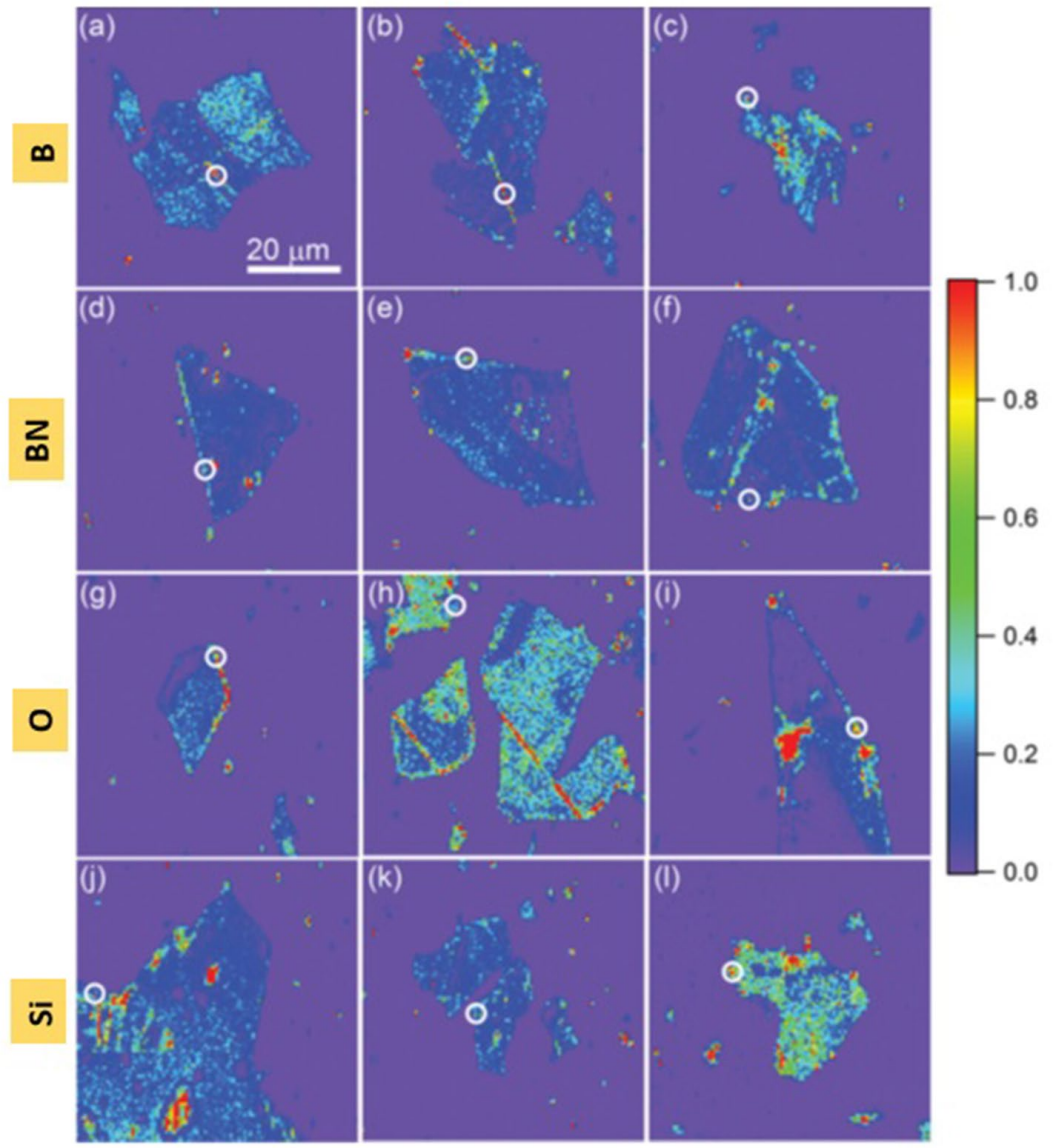
Localization of emitters at exfoliated multilayer hBN flake edges^98^. Confocal PL maps of exfoliated multilayer hBN flakes in which stable emitters (white circles) are detected at flake edges after ion implantation followed by annealing. (**a–c**) boron implanted, (**d**–**f**) boron-nitrogen complex implanted, (**g**–**i**) oxygen implanted, (**j**–**l**) silicon implanted flakes. Large bright luminescence observed away from flake edges does not exhibit photon antibunching as in map (**i**).

Other robust methodologies determined to fabricate quantum emitters in CVD grown multilayer and exfoliated multilayer hBN flakes are electron beam irradiation, laser irradiation, ion implantation^65,98^ and Ar plasma etching^66^ and their corresponding processing details were listed in Table 7. The stable emitters localize at flake edges after ion implantation and subsequent annealing is shown in Fig. 6 and the comparison for photostability of emitters formed due to various ion implantations and activated through only annealing is shown in Table 8.

**Table 7:** Sample processing details of various quantum emitter fabrication techniques.

Quantum emitter fabrication techniques	Details	References
Electron beam irradiation	Energy: 15 keV, fluence: 5 × 10^18^ (e/cm^2^), temp: RT	^65,98^
Laser irradiation	λ: 515 nm, pulse width: 230 fs, number of pulse: 1, temp: RT	
Ion implantation	Energy: 50–70 keV, fluence: 10^10^ ions/cm^2^, temp: RT	
Ar plasma etching	Power: 200 W, pressure: 180 mTorr for 2 min, temp: RT	^66^

The details of energy E, electron fluence (electron beam irradiation), ion fluence (ion implantation), Wavelength λ and Power P optimized for sample processing and all techniques were performed at room temperature.

**Table 8:** Comparison table for emitter formation between various ion implantations and unimplanted (only initial annealing) flakes.

Exfoliated hBN sample	Number of flakes examined	Number of emitters found	Remarks	Reference
B irradiated	7	10	The number of stable emitters formation probability is more in ion implanted samples than the sample which has undergone only annealing	^98^
BN irradiated	12	10 (3 were blinking)		
O irradiated	12	10		
Si irradiated	8	11 (3 were blinking)		
Only annealed reference sample	16	7 (3 were blinking)		

The information in the table conforms that stable emitter formation probability is more in ion implanted samples than the reference sample (only annealed). Emitters formed by ion implantation followed by annealing exhibits absolute photostability whereas 40% of emitters formed through only initial annealing exhibit severe blinking and eventual bleaching.

Quantum emitters are engineered in CVD grown multilayer hBN and exfoliated multilayer hBN flakes, by performing stable emitter fabrication techniques as described in Table 8.

To date, no supreme emitter fabrication techniques were experimentally demonstrated for bulk (except annealing for emitter activation) and monolayer hBN and no annealing is performed to activate emitters in monolayer hBN as^64,99^ surface states are often unstable and modifies upon annealing. An important analysis from emitter formation and activation process are listed in Table 9.

**Table 9:** Analysis from emitter formation and emitter activation process.

Analysis from emitter formation and activation process	References
Electron beam irradiation, Laser irradiation, Ion implantation and Ar plasma etching generates new emitters	^66,98^
High temperature annealing only for emitter activation	^98^
Emitter formation probability is more in Laser irradiation as laser ablation breaks the samples into small fragments results in more number of emitters	
Samples are annealed after ion implantation and laser irradiation, which creates significant damage to hBN lattice that partly recovers during annealing	
Annealing of samples is not require after electron beam irradiation, as it is a subtler process reforms the lattice chemically with minimal damage	
Ion implantation has little influence on defect formation probability, indicates bombarding of ions introduces vacancies and activates intrinsic point defects already present, than introducing foreign fluorescent defects	^98,100^
Ion implantation increases stability of emitters and the hypothesis that ion implantation provides sufficient kinetic energy to eliminate some of the trapped species in the vicinity of emitter which leads to eliminate blinking of emitter	^98,101^

The analysis of emitter formation and emitter activation due to various emitter fabrication techniques, their hypothesis and important facts related to sequential annealing.

In exfoliated multilayer hBN flakes, Ar plasma etching and subsequent annealing in argon produces emitters with emission wavelength greater than 700 nm^66^ and cathodoluminescence analysis of exfoliated multilayer flakes found single photon emission in the UV region^63^ with a ZPL at around ~ 302 nm.

## Photophysics of hBN quantum emitters

In traditional materials like diamond and silicon carbide, colour centres have similar spectral properties in both bulk and nano dimensional forms. But, in case of van der Waals crystals, the optical properties of 2D materials are different from their bulk^102^ structures. Due to this reason, we present the quantum emission properties of hBN material in bulk, multi and mono layer form.

### Optical characterization of hBN quantum emitters

#### Bulk hBN

**Figure 7:**
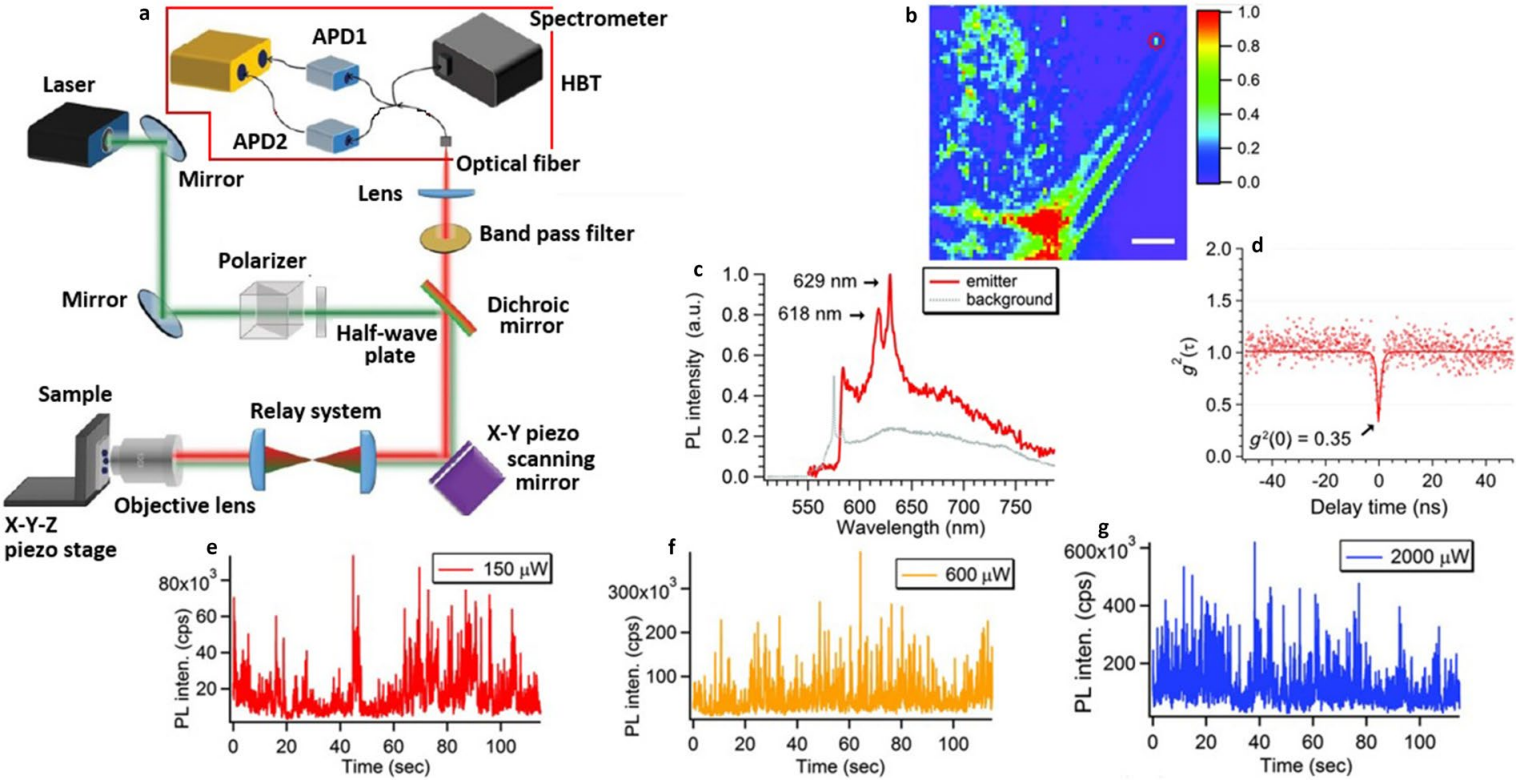
Schematic of confocal photoluminescence (PL) set-up coupled to HBT for optical characterization, optical characterization of emitter in bulk hBN and its blinking behaviour at elevated excitation powers^65,96^. (**a**) Complete optical characterization setup (Confocal PL setup coupled to HBT), photoluminescence setup for identifying and analyzing the emitters. HBT interferometer for second order autocorrelation measurements. (**b**) Confocal photoluminescence map of bulk hBN, obtained by 532 nm CW laser excitation and the scale bar indicates 10 μm. (**c**) Photoluminescence spectrum of isolated emitter (532 nm CW laser excitation) represented by solid red trace along with background spectrum obtained from the region adjacent to emitter represented by dotted grey trace. (**d**) Second order autocorrelation measurements $g^{2} \left( 0 \right)$ ~ 0.35, performed by using HBT interferometry at one of the ZPL. The obtained experimental data (red dots) is not background corrected and the dip of the curve below 0.5 indicates the single photon emission nature of defect. The fluorescence intensity plots of emitter (detected for 675 nm excitation) recorded as a function of time at excitation powers (**e**) 150 μW, (**f**) 600 μW and (**g**) 2000 μW. Blinking of defect is clearly visible for all the investigated powers.

Optical characterization of quantum emitters in bulk hBN^96^ was performed by complete optical characterization setup (Confocal photoluminescence (PL) setup couple to HBT interferometer) as shown in Fig. 7a.

The PL map of bulk hBN shown in Fig. 7b represents an isolated single photon emitter (circled in red colour) along with other ensemble emissions. The corresponding PL spectrum and second order correlation measurements were shown in Fig. 7c, d.

The red dots in second order autocorrelation graph, shown in Fig. 7d, represent experimental data and the solid red line is a fit by using a three level model:

1 $$g^{2} \left( \tau \right) = 1 - \left( {1 + a} \right)e^{{\frac{ - \left| \tau \right|}{{\tau^{1} }}}} + ae^{{\frac{ - \left| \tau \right|}{{\tau^{2} }}}}$$

where $\tau^{1}$ = lifetime of excited state, $\tau^{2}$ = lifetime of metastable state and **a** = bunching factor.

The emission from the defect bleaches after several minutes of excitation with 532 nm laser source. To obtain additional insight about luminescent point defect detection and its bleaching affect, the optical characterization was performed by replacing the 532 nm laser with another excitation laser of long wavelength (675 nm).

As similar to the emitter highlighted in Fig. 7b, an isolated emission with ZPL 760 nm, which satisfying the single photon emission characteristics is found. However, Bleaching is not observed for 675 nm excitation but another effect blinking is observed. This blinking behaviour of defect is clearly observed, for different excitation powers as shown in Fig. 7e–g.

Although, the emitters in bulk crystals are brightest and most stable^103–105^ but has the disadvantages as listed in Table 10, which can be overcome by using atomically thin 2D hBN.

**Table 10:**
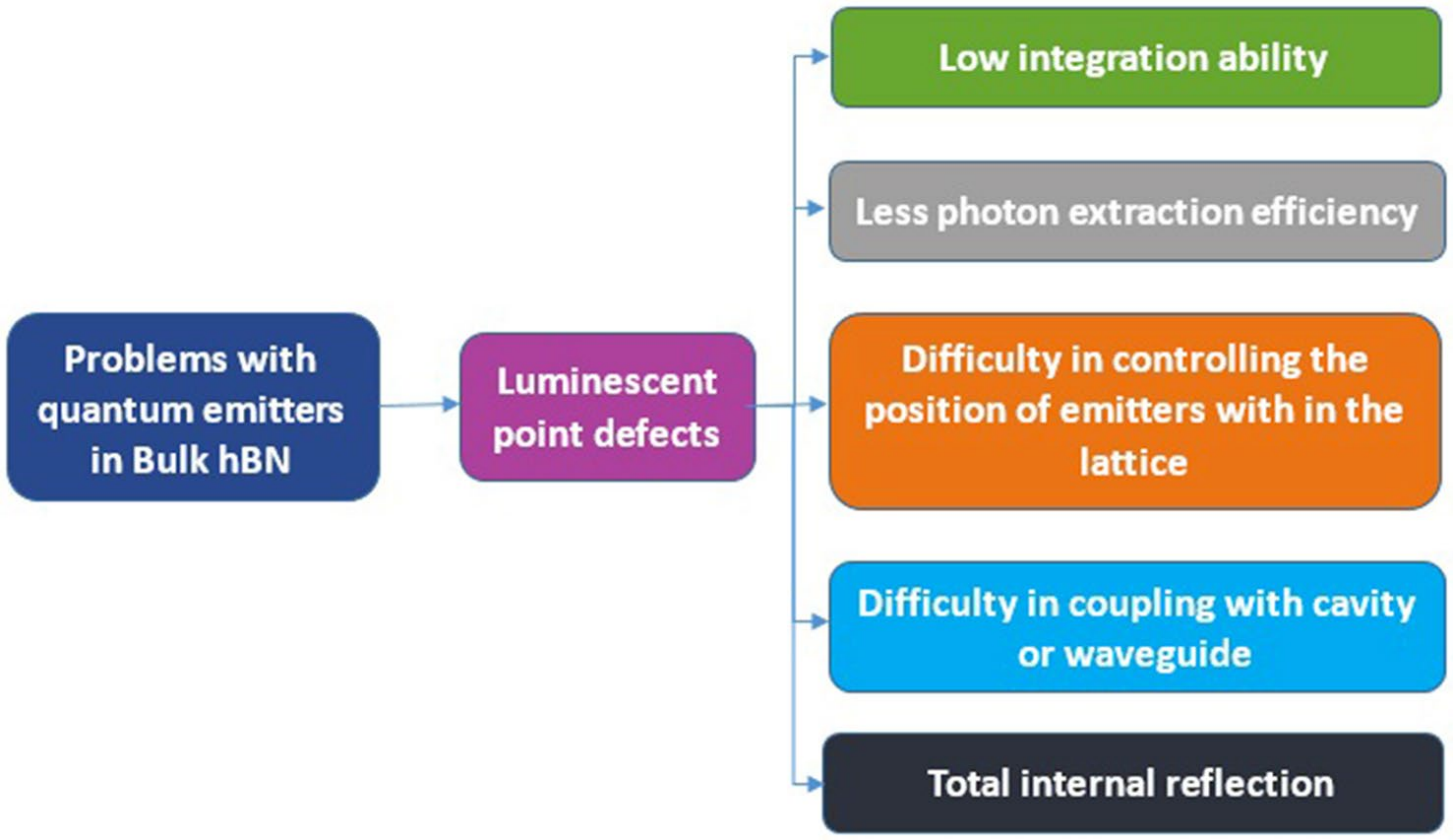
The five major problems associated with quantum emitters in bulk hBN.

Specifically emitters reported in 2D hBN has PL spectra, photostability and photodynamic characteristics which are significantly different from their bulk counterpart^96,102^.

#### CVD grown and exfoliated multi, single hBN layers

Optical characterization of emitters from CVD grown hBN multi, single layer and exfoliated multilayer flakes were analyzed and it was noted that their PL spectrum exhibiting ZPL at ~ 580 nm to 623 nm.

The emission lines in multilayer are narrower than monolayer as observed in ref^64^ and the emitter in hBN multilayer exhibits a stable fluorescence without any blinking or bleaching (at excitation power: 1mW, Time: 10 min). But in case of hBN monolayer the emitter blink and bleach for continuous excitation.

The creation and characterization of single photon emitters in a suspended, single crystal hBN film and free from substrate interactions were recently studied as in ref^106^. The emitters detected exhibits ZPL range from 550 to 700 nm with better single photon purity.

A significant difference in brightness was observed for the emitters detected in freely suspended hBN film compared to the emitters detected in substrate supported hBN, which provides a breakthrough advances in fabricating quantum photonic circuits.

Fluorescence measurements from traditional ensemble emitters were limited due to heterogeneity of emitters and individual defect-by defect studies are impractical. New techniques were proposed to quickly and accurately characterize the emission from ensemble emitters as experimented in ref^107^.

### Optical stability of hBN quantum emitters towards wide range of temperatures and various annealing environments

Quantum emitters in hBN multilayer exhibits absolute thermal stability, optically stable even when operated upto 800 K^54^, capable to withstand aggressive annealing treatments in both oxidizing and reducing environments without any change in spectral properties^65^.

**Figure 8:**
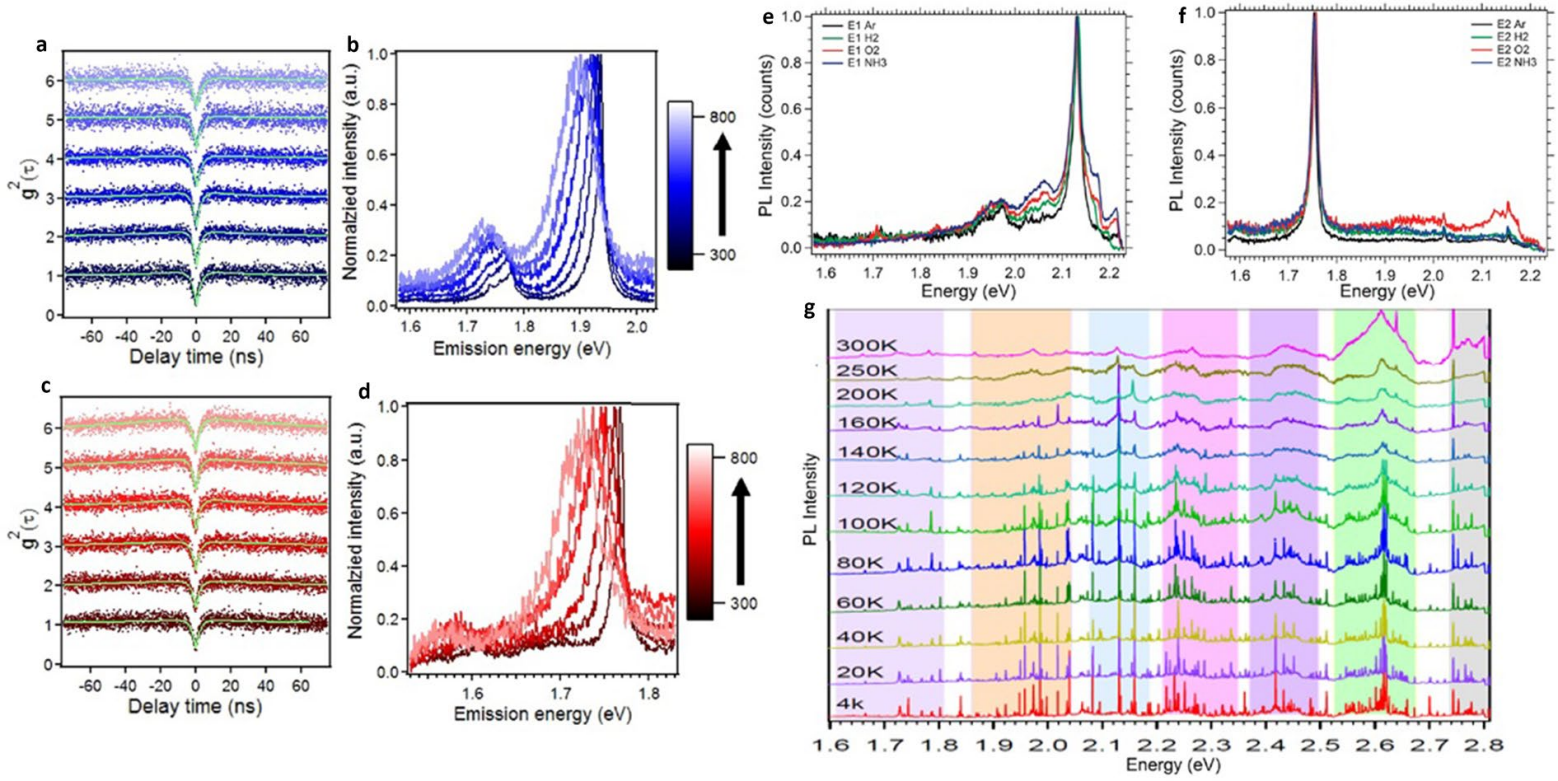
Stability of emitters towards temperature variations and different annealing environments and PL spectrum of a hBN quantum emitter observed from cryogenic to room temperature^54,55,65^. Characterization of emitters during heating phase with temperature increments of 100 K during 300 − 800 K thermal cycle. (**a**–**d**) Second order correlation measurements and corresponding PL spectrum of emitter having ZPLs ~ 1.94 eV and ~ 1.75 eV respectively. (**e**, **f**) PL spectrum recorded for two different emitters after each annealing treatment in argon (initial annealing), hydrogen, oxygen, and ammonia respectively. There is no change in the PL spectrum after each annealing treatment. (**g**) The PL spectrum of hBN emitter observed from 4 to 300 K. The Width of the ZPL narrows with decrease in temperature and vice versa.

The emitters’ stability towards temperature variation, annealing treatments in various gaseous environments is shown in Fig. 8a–f and in particular the variation in PL spectrum^55^ of an emitter observed from cryogenic to room temperature is shown in Fig. 8g. An important analysis related to nature of emitters obtained from various annealing environments is summarized in Table 11.

**Table 11:** Analysis of nature of emitters from various annealing environments.

Analysis of nature of emitters from various annealing environments	References
Luminescent defect might have a vacancy in its crystallographic structure, whose formation probability proportional to annealing temperature	^65,108^
Emitters are likely to be neutrally charged because annealing in hydrogen environment would expect to modify the negative charged state to neutral charge state	^109^
Emitters which are stable even after annealing might not be the surface states (observed in some TMDs) because surface states are unstable and can be easily modified by annealing in various reactive environments	^65,99^
Each annealing step creates some new emitters and quench some emitters and this quenching occurs for unstable emitters possibly located in topmost layers or edges of hBN flakes, sensitive to annealing	^65^

The analysis of nature of emitters and its formation, crystallographic structure, charge states, location, stability of emitters and role of annealing temperature.

### Emission wavelength range of hBN quantum emitters

Quantum emitters in hBN multilayers exhibit a broad range of UV to near IR single photon emission^63–66^ as listed in Table 1, which is in contrast to colour centers and quantum dots (emission at a particular wavelength or over a narrow spectral range).

Emitters in hBN multilayer are classified into two groups based on Zero phonon line (ZPL) and phonon sideband (PSB) shapes. Emitters in group 1 have broad ZPLs along with PSBs and energies ranging from 576 nm (2.15 eV) to 652 nm (1.90 eV). Emitters in group 2 have narrow symmetric ZPLs with weak PSBs and energies ranging from 681 nm (1.82 eV) to 762 nm (1.63 eV).

In a survey of ~ 40 emitters, 70% belongs to group 1 and rest 30% of emitters belongs to group 2. The variation in ZPL of emitters in both the groups is due to variations in local strain and dielectric environment^65^.

**Figure 9:**
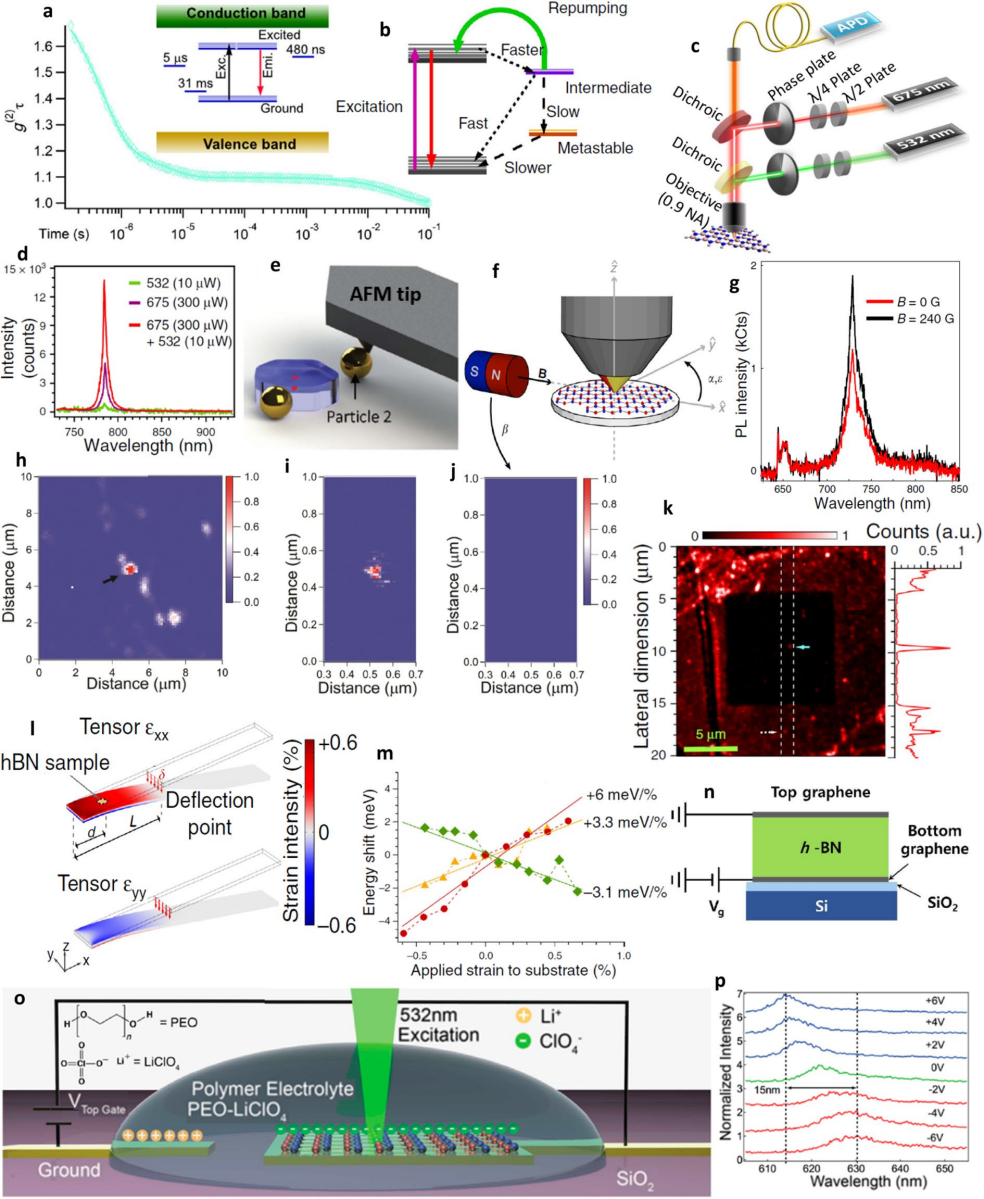
Correlation measurements over a long time scale, pictographic representation of emitter level structure^96^, schematics of two laser excitation^113^, plasmonic coupling^114^, external strain^78^, electric^118^ and magnetic field^57^ inducements and ionic liquid devices^97^, off and on-resonant excitation^116^ PL maps and corresponding PL spectrums of quantum emission enhancement techniques. (**a**) Second order autocorrelation measurements $g^{2} \left( 0 \right)$ performed over a long time scale, three exponential components best fits for autocorrelation curve which indicates the presence of metastable states between ground and excited states. Inset shows the presence of three metastable states with lifetimes of 5 µs, 31 ms and 480 ns. (**b**) Schematic representation of fast decaying intermediate and a long-lived metastable states and reversion of emitters from intermediate to excited state by repumping technique. (**c**) Schematic of two laser beam excitation of different wavelengths. (**d**) PL intensity of emitter for single lasers 532 nm, 675 nm and two lasers (532 nm + 675 nm) excitation. (**e**) A single gold nanosphere is coupled to emitter in flake and a second gold nanosphere is made to couple by using an AFM tip in order to form plasmonic cavity. (**f**) Schematic representation of horizontally induced magnetic field to the sample, which is under optical excitation. (**g**) Variation in fluorescence intensity of emitter due to applied magnetic field. (**h**–**i**) The PL maps of emitter under off and on-resonant excitations respectively, in which quantum emission is indicated by black arrow, the other white areas represent the emission from background (unwanted), which is not observed in on-resonant excitation PL map. (**j**) No PL signal is observed when resonant excitation was detuned (reduced) by 2 nm. (**k**) The PL map of irradiated area (He + ion implanted area, followed by annealing with argon) which exhibits a dark region indicating the reduction of background fluorescence compared to non-irradiated area and corresponding PL spectrum of white dashed area is shown in right panel. (**l**) Tensile (compressive) strain applied by a polycarbonate (PC) beam in which vertical force is applied to a free edge and colour represents the possible strain intensity.(d = distance between the fixed edge and hBN sample, L = distance between fixed edge and deflection point and $\delta$ = beam deflection). (**m**) ZPL energy shits as a function of applied strain for three different emitters and linear fit indicates overall strain induced ZPL energy shift ranges from − 3 to 6 meV/%. (**n**) Schematic representation of multilayer hBN sandwiched between two graphene electrodes (induce electric field) for stark tuning of emitters embedded in hBN. (**o**) Schematic representation of LPCVD grown hBN (transferred on gold electrode using PMMA assisted transfer) placed in an ionic liquid device. The gate voltage is applied to second electrode. (**p**) PL spectrum of emitter around 622 nm under no bias, blue and red-shifted by 15 nm for ± 6 V gate voltages respectively.

The autocorrelation function measurements over a long time scale ^65,96^ of 0.1 s as shown in Fig. 9a provides an information about the presence of metastable states with decay times^110–112^. The presence of fewer metastable states with shorter life time makes the emitter brighter than the emitter having more metastable states with long lifetimes.

## Modulation of emission characteristics of hBN quantum emitters (mechanical, optical, magnetic and electrical tuning)

However, robust, optically strong and thermally stable single photon emitters are detected in hBN, these emitters faces the limitations of moderate emitter brightness (fluorescence intensity), non-ideal single photon purity, more emitter lifetime, spectral diffusion at cryogenic temperatures and inhomogeneous emission distribution over a large spectral band. These limitations create a central problem for fabricating identical single photon emitters with ideal characteristics, which strengthen to fabricate the efficient quantum applications.

Many new techniques are proposed and proved to overcome these limitations and enhance the emission characteristics of hBN quantum emitters as explained below:

### Simultaneous excitation with two laser beams of different wavelengths

Excitation of hBN quantum emitters with single laser (either with longer wavelength (675 nm) and high power (300 µW) or with mid-wavelength (532 nm) and low power (10 µW)), exhibits the photoluminescence measurements having moderate or less fluorescence intensity and photon emission rate.

Autocorrelation measurements of quantum emitters (over a long time scale) revealed existence of a fast decaying intermediate state and a long-lived metastable state within the transition energy gap as shown in Fig. 9a and the electron decay from this long-lived metastable state leads to poor antibunching factor, low photon emission rate and needs high laser excitation power (to saturate emission intensity).

By employing simultaneous both red laser (675 nm and 300 µW) and green laser (532 nm and 10 µW) excitations^113^ as shown in Fig. 9c, the enhancement in fluorescence intensity was observed due to certainty that the electrons are reverted from long-lived intermediate state to excited state, which leads to repopulation of excited state (terminating the electron decay from long-lived metastable state) as shown in Fig. 9b and the corresponding enhancement in emitter fluorescence intensity is shown in Fig. 9d.

The intensified photo physical characteristics of emitter due to two laser excitation in comparison with single laser excitation were listed in Table 13. It is noted that there is an enhancement in emitter fluorescence intensity and reduction of excitation saturation power, which are desirable, for two laser excitation in comparison to single laser excitation technique.

### Enhancing quantum emission due to coupling with plasmonic nanoparticle or via external magnetic field inducement

#### Coupling with plasmonic nanoparticle

Another superior technique to enhance the quantum emitter characteristics rather than simultaneous exciting with two laser beams is coupling the quantum emitters with plasmonic nanoparticles like gold, silver and platinum etc.

Overall photon emission rate enhancement is made possible by combination of both excitation enhancement and spontaneous enhancement rate. Coupling the gold nanospheres to the emitters in exfoliated flake edges as observed in ref^114^, enhances the excitation rate due to surface Plasmon resonance and spontaneous emission rate enhancement by Purcell effect (enhancement by environment).

Using AFM tip, the gold nanospheres are positioned to close proximity of emitters as shown in Fig. 9e, made to align with transition dipole angle of emitter in order to increase the plasmonic coupling effect and this transition dipole angle was deduced from flake orientation. The enhancement in quantum emitter characteristics due to single and double nanospheres coupling were listed in second row of the Table 13.

#### External magnetic field inducement

An effective alternate to plasmonic coupling enhancement is by inducing external magnetic field.

It is a known fact that electronic structure of defects in wide band semiconductors possess both electron un-occupied and occupied states in between the conduction and valence band edges of the energy gap. These occupied electrons exists in paramagnetic form (possibly with up ↑ or down ↓ spins). By inducing external magnetic field, these occupied energy states split up due to Zeeman effect (let's say one occupied state with a paramagnetic electron split up into two higher and lower energy states with energy difference (ΔE) and this paramagnetic electron prefers to sit in lower energy state).

When this lower energy paramagnetic electron absorbs a photon with energy = ΔE, then it jumps to higher energy state and emits the photon (with same or different energy) while relaxing back to lower energy state. This spin-dependent inter-system crossing (ISC) transition found to exhibit an anisotropic fluorescence response for applied magnetic field as experimented in ref^57^.

The schematic representation of inducing external magnetic field to the hBN film and simultaneous optical excitation is shown in Fig. 9f, where β represents angle made by horizontally applied external magnetic field w.r.t ${\hat{\text{x}}}$ plane and α (ε) represents absorption (emission) dipole w.r.t $\widehat{{\text{x}}} - \widehat{{\text{y}}}$ plane.

The variation in PL spectrum with and without magnetic field is shown in Fig. 9g. The quantum emitter fluorescence emission found to increase monotonically for particular combination of applied magnetic field angle and absorption dipole angles as listed in Table 12.

**Table 12:** Higher fluorescence quantum emission observed for particular combination of applied magnetic field and light absorption dipole angles.

Horizontally applied external magnetic field angle (β) w.r.t ${\hat{\text{x}}}$ plane	Absorption dipole angle (α) w.r.t $\widehat{{\text{x}}} - \widehat{{\text{y}}}$ plane	Reference
β = 0°	α = 90°	^57^
β = 45°	α = 45° and 90°	
β = 90°	α = 0° and 90°	

Monotonical increase in fluorescence quantum emission is observed for β = 0° with applied constant magnetic field strength (B) = 890 Gauss and for both β = 45° and 90°, increase in fluorescence quantum emission is examined by varying magnetic field strength (B) from 0 to 400 Gauss.

### Resonant excitation technique

At cryogenic temperatures, the ZPL shape of emitters was influenced and broadened due to spectral diffusion^115^, which leads to erroneous ZPL measurements.

This spectral diffusion is observed in off-resonant excitation (electron is excited to higher vibronic energy levels of excited state and this excited electron should vibrationally relax to minimum energy level of excited state and followed by spontaneous emission to emit a single photon). The control of ZPL broadening (even when emitters experience spectral diffusion) is observed in on-resonant excitation^116^ (electron is excited directly to minimum of excited state and then follows spontaneous emission).

This off- resonant excitation occurs when excitation energy (*hf*) is more than luminescent point defect ZPL energy (ΔE_ZPL_) and on-resonant excitation occurs when *hf* = ΔE_ZPL_. The controlled photophysical characteristics of emitters due to on resonant excitation is shown in third row of the Table 13 and the variation in PL maps due to Off and On resonant excitations is shown in Fig. 9h–j.

**Table 13:** Intensified photophysical characteristics of emitters due to three different enhancement techniques. Enhancement in fluorescence intensity and reduction of excitation saturation power for two laser excitation technique has shown in first row; increase in photon emission rate and controlled fluorescence lifetime due to two nanospheres plasmonic coupling. Modulated ZPL (wavelength and width); enhanced single photon purity and controlled fluorescence lifetime due to on-resonant excitation is shown in third row.

Enhancement techniques	Excitation and emission wavelength (ZPL width)	Fluorescence intensity and saturation power	Photon emission rate	Lifetime	g^2^ (0)	References
**Laser excitation technique**						
Two laser excitation	675 nm + 532 nm and 778 nm (ZPL width N/A)	∼ 14 × 10^3^ Hz and (1.5–3) mW	N/A	N/A	0.25	^113^
Single laser excitation	675 nm and 778 nm (ZPL width N/A)	∼ 5 × 10^3^ Hz and ∼ 14 mW				
**Plasmonic coupling**						
Single nanosphere	532 nm and 578 nm (ZPL width N/A)	N/A	2.89 × 10^6^ Hz	2.68 ns	0.26	^114^
Two nanosphere			5.79 × 10^6^ Hz	1.54 ns	0.31	
**Resonant excitation**						
Off-resonant excitation	700 nm and 766.8 nm (ZPL width ∼ 25 GHz)	1 × 10^6^ Hz	N/A	∼ 3 ns	0.16 ± 0.01	^116^
On-resonant excitation	700 nm and 766.186 nm (ZPL width ∼ 6.3 ± 0.3 GHz)	N/A		∼ 0.87 ns	0.11 ± 0.04	

### Enhancement of single photon purity

Second order correlation values found to vary around 0.5 and this deviation from $g^{2} \left( 0 \right)$ = 0 (ideal value) is due to high residual background emission, which leads to decrease in single photon purity.

Emitters found in the He^+^ ion implanted area with subsequent annealing with argon (shown as a dark region) exhibits correlation value $g^{2} \left( 0 \right)$ = 0.077 (indicated by blue arrow), reduced by five-fold^78^ (indicating the reduction of background fluorescence) compared to the emitters found in non-irradiated area exhibiting $g^{2} \left( 0 \right)$ = 0.263 (indicated by white dashed arrow) as shown in Fig. 9k. The corresponding PL spectrum is shown in right to PL map.

### Tuning the quantum emission through external strain inducement or external electric field inducement or through ionic liquid devices

Emitters in hBN multilayers exhibit different emission energies (ZPL) over a large spectral band (inhomogeneous spectral distribution)^65^ due to variations in local strain and dielectric environment, which creates a central problem for fabricating identical single photon emitters.

Tuning the quantum emitters (having uneven ZPL emission energies), through external strain inducement or through external electric field via stark effect or electrostatic tuning through ionic liquid devices, leads to increase the probability of multiple uneven emitters (different ZPL energies) to have the same emission energy (ZPL), which further tends to fabricate identical quantum emitters with necessary emission wavelength.

#### External strain inducement

Controllable external strain can be induced to the emitters in hBN film by placing on a 1.5 mm thick bendable polycarbonate (PC) beam^78^ as shown in Fig. 9l and corresponding ZPL energy shifts of emitters due to variation in applied strain is shown in Fig. 9m. Tunability range observed for three different emitters (with different ZPL energies) are −3.1 meV/% (green), + 3.3 meV/% (yellow) and + 6 meV/% (red) respectively.

Similar experimental approach was observed in ref^117^ for deterministic single photon emitter formation by a combination of nanoscale strain engineering and charge trapping.

#### External electric field inducement

Quantum emitters in hBN are expected to lie within the plane, which creates an in-plane dipole. By inducing an out-of-plane electric field through graphene gates, electrical tuning of quantum emission (via stark effect) from luminescent point defects hosts in hBN was observed in ref^118^. The schematic representation of multilayer hBN between top and bottom graphene electrodes (for electric field inducement) is shown in Fig. 9n.

#### Electrostatic tuning of quantum emission through ionic liquid devices

The quantum emission from luminescent point defects in hBN can also be tuned by placing the hBN sample in ionic liquid devices^97^ as shown in Fig. 9o, in which poly (ethylene oxide) mixed with lithium perchlorate (PEO: Li-ClO4) is used as an electrolyte.

By applying positive (negative) gate voltages, the ZPL emission is found to be blue shifted (red shifted) by around 15 nm as shown in Fig. 9p. Tuned ZPL emissions are found to be stable and does not exhibit spectral diffusion.

### Other recent advances in modulation of emission from 2D hBN quantum emitters

**Figure 10:**
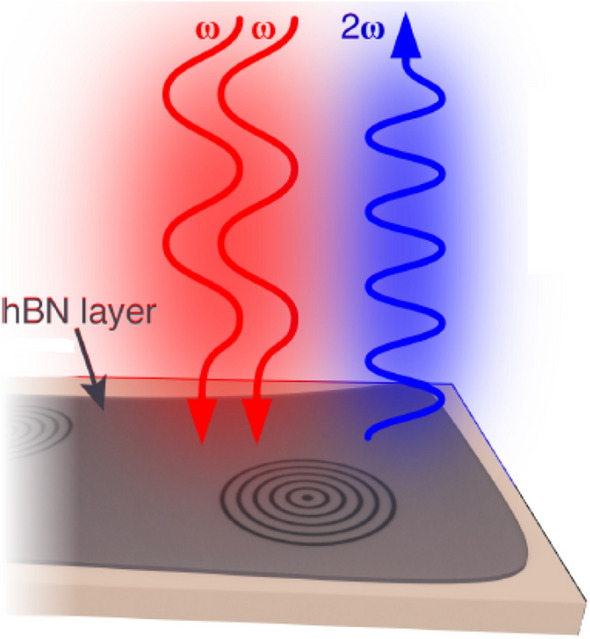
Schematic representation the SHG from a few-layered hBN film on a CBG. Schematic representation of multilayer hBN coupled to circular Bragg grating photonic microstructure and found to exhibit second harmonic generation by absorbing two photons of same frequency and generating a single photon of twice the frequency absorbed. *Figure* a*dapted with permission from ref. [Bernhardt, N *et al*., "Large few-layer hexagonal boron nitride flakes for nonlinear optics", Optics Letters, 46(3), p.564, 2021], The optical society.*

Contemporary exploration on hBN integration with photonic microstructures like circular Bragg grating (CBG) was found to be eminent and rooted out the second harmonic generation^119^ (absorbs two photons of same frequency and generates a single photon of twice the frequency absorbed) in exfoliated multilayer hBN as shown in Fig. 10. Some of the investigative explorations probe that this second harmonic generation (SHG) could be associated to defects^120^.

Novel techniques to enhance the quantum emission from hBN single photon sources is by coupling the quantum emitters with photonic crystal cavities from silicon nitride (Si_3_N_4_)^121^ and Al nano-antenna^122^, which revealed a 6-fold and 10 to 15 fold enhancement in photoluminescence measurements of a hBN quantum emitter at room temperature respectively.

Due to high transmittance and poor reflectance of monolayer hBN, it was found to be invisible under white light which leads to utmost difficulties in experimental works like transferring methods and fabrication of 2D heterostructures.

It was Serendipity, that hBN was found to enhance the contrast on transparent substrates^123^ (polymer based interfacial layer on a polydimethylsiloxane (PDMS) substrate) and similar investigation was performed on other 2D TMDs and disseminate that TMDs exhibit significant difference in contrast on transparent substrates than opaque substrates.

The simulation results also reveal that contrast of hBN is high under shorter wavelength light than longer wavelength. Finally it was negotiated that selecting an appropriate substrate for optical experimentation is very crucial.

Tuning of quantum emission spectrum is found to feasible with externally applied tensile strain and present day a record tuning of 65 meV^124^ was observed in hBN layers.

## Prediction of emitter defect structures and applications of quantum emitters

### Prediction of defect structures using first principle calculations

**Figure 11:**
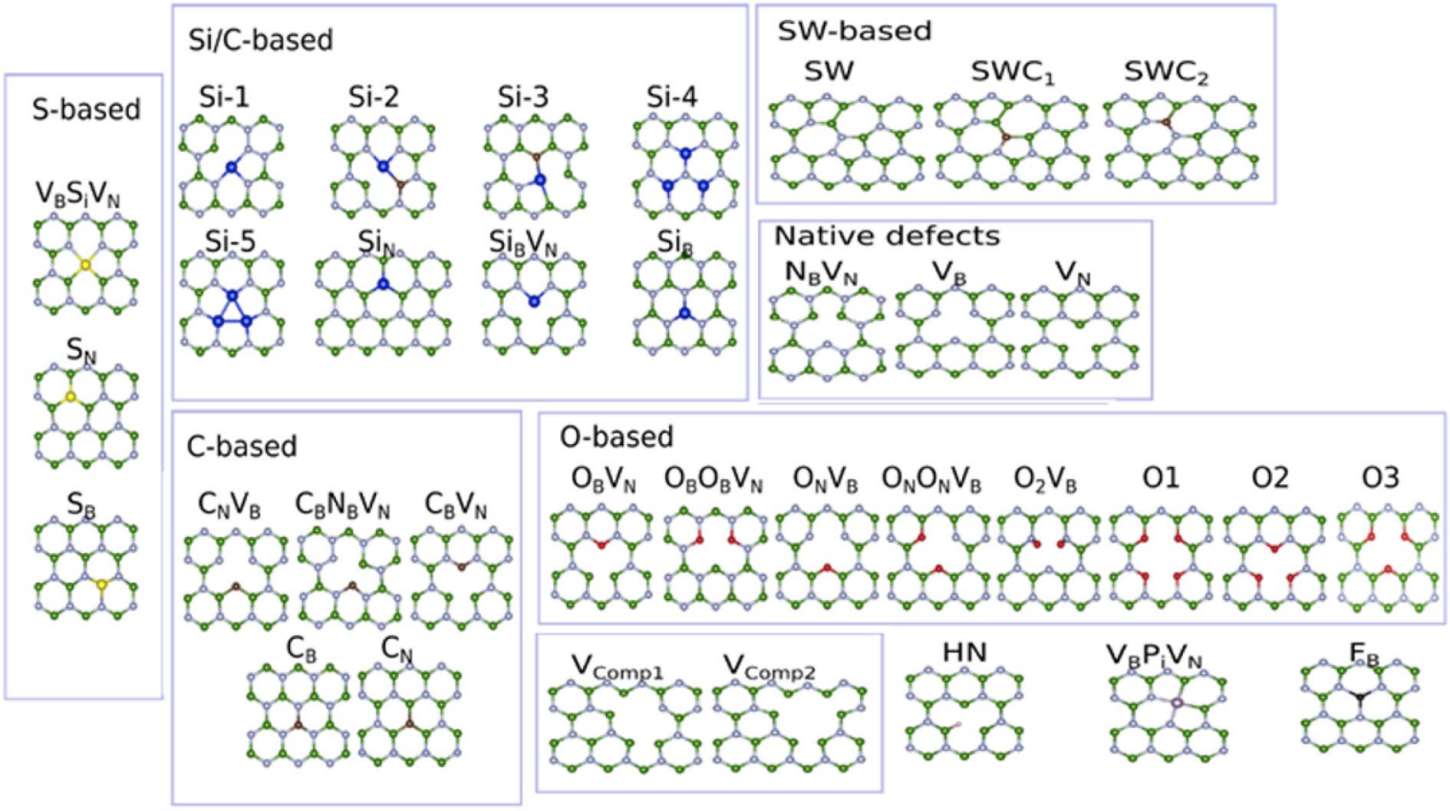
Atomic structures of possible luminescent point defects for hBN quantum emitters^62^. Atomic structures of 35 different possible hBN defects due to likely conditions. Defects group such as Si/C-defects, Stone–Wales defects (SWC_N_), C-based defects, O-based defects, native defects, S-based defects, complex vacancy defects (VCompX) and few other defects were considered. Legend: white spheres represent nitrogen atoms, green spheres (boron), red spheres (oxygen), blue spheres (silicon), brown spheres (carbon), yellow spheres (sulphur), black spheres (fluorine), silver spheres (phosphorus),small white spheres (hydrogen).

A list of 35 different possible luminescent point defects of hBN (based on most likely forming impurities incorporate during hBN growth and while annealing on substrate), as shown in Fig. 11, were examined by Density Functional Theory (DFT) calculations using Spanish Initiative for Electronic Simulations with Thousands of Atoms (SIESTA)^125^ and Vienna Ab initio Simulation Package (VASP)^126^ on the basis of generalized gradient approximation by Perdew, Burke and Ernzerhof (PBE) functionals^127^.

Based on experimental observations, three main conditions, as shown in Table 14, were applied to authenticate a luminescent point defect as an effective potential quantum emitter.

**Table 14:** Conditions to authenticate a luminescent point defect as an effective potential quantum emitter.

Three main conditions to authenticate a luminescent point defect as potential quantum emitter	References
The energy levels of luminescent point defects must be located within the bandgap and none of the levels should be present within or near to bulk bands, which represents that these point defects are thermally stable against annealing and higher temperatures, which is the characteristic of an essential quantum emitter	^128^
Theoretically calculated emission energies of luminescent point defects must be consistent with observed ZPL energies of quantum emitters in experimental studies	^129,130^
Luminescent point defects must exhibit polarized excitation and emission in optical spectrum (simulated), as this polarization phenomena was observed in quantum emitters during experimental analysis	^62^

Among the 35 luminescent point defects, N_B_V_N_, O_B_O_B_V_N_ and C_B_V_N_ are the defect structures found to satisfy the rules listed in Table 14 and their corresponding ZPL energies are 2.01 eV, 1.85 eV and 1.33 eV respectively (obtained through VASP calculations).

**Figure 12:**
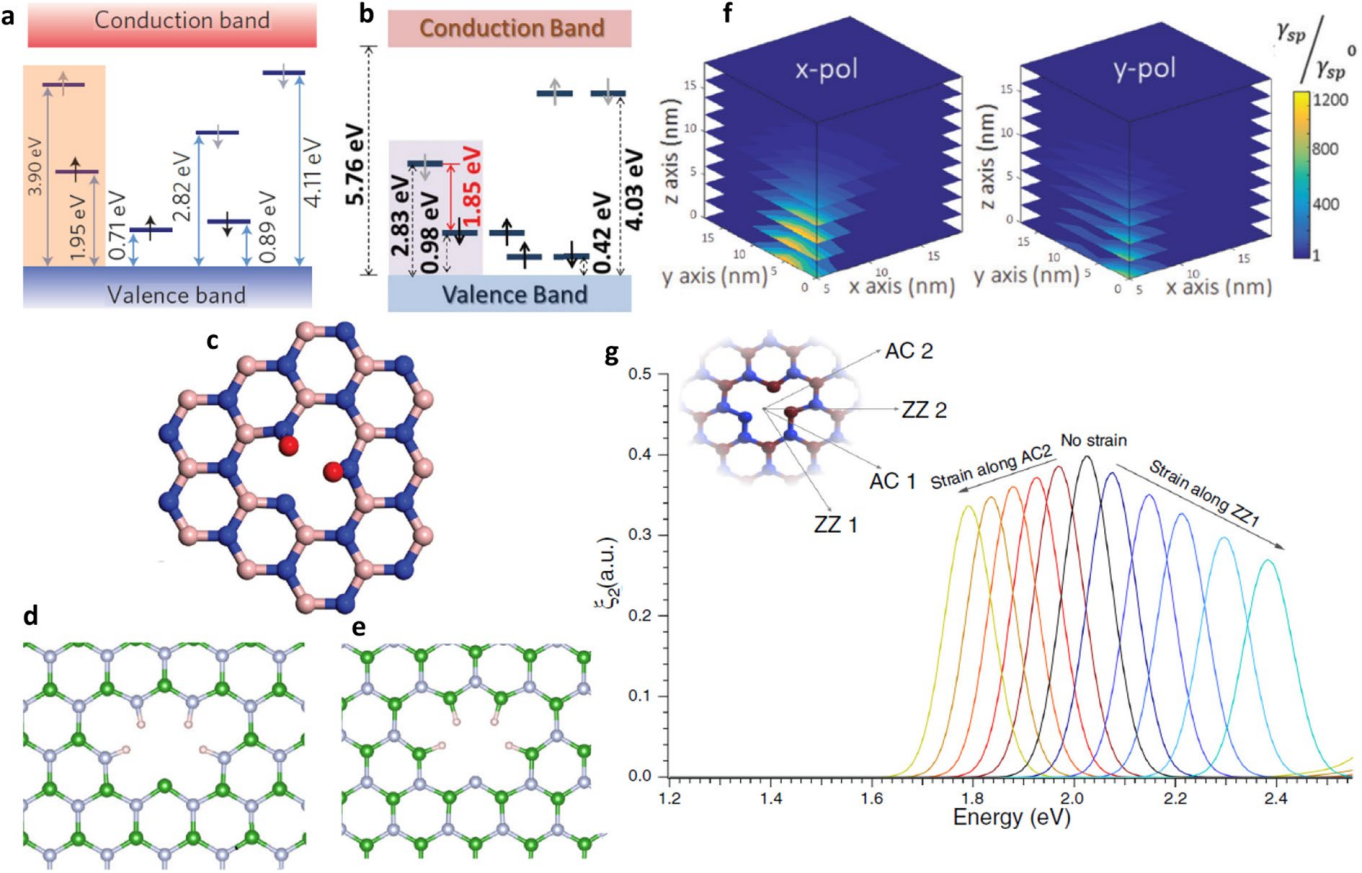
Simulated electronic structures of N_B_V_N_^64^ and V_B_O_2_^66^ defects, schematic representation of V_B_O_2_ defect in hBN monolayer, boron and nitrogen dangling bonds^131^, coupling of gold nanosphere to emitter in hBN multilayer flake^114^, Strain directions and spectral tuning of hBN quantum emitter and^78^. (**a**, **b**) Electronic structures of N_B_V_N_ and V_B_O_2_ defect, in which 1.95 eV transition (transition from a ground state at 1.95 eV to an excited state located at 3.90 eV) and 1.85 eV transition (transition from a ground state at 0.98 eV to an excited state located at 2.83 eV) were highlighted, consistent with experimental studies and Only spin-preserving transitions are assumed and occupied and unoccupied states are represented by black and grey arrows. (**c–e**) Schematic representation of V_B_O_2_ defect (Boron vacancy with two oxygen atoms (red colour)), Boron (green) and Nitrogen (grey) dangling bonds with hydrogen (white) passivated respectively. (**f**) Spontaneous emission enhancement (γ_sp_/γ^0^_sp_) due to defect coupling with gold nanospheres. (**g**) Effect of strain on N_B_V_N_ defect structure.

The Emitters detected in experimental studies (emission in visible region) whose ZPL is around ~ 2 eV, predicted to have N_B_V_N_^64^ defect structure and simulated (using VASP) electronic structure of N_B_V_N_ defect in hBN monolayer is shown in Fig. 12a.

The electronic structure of V_B_O_2_ defect (obtained using VASP simulations) in hBN monolayer is shown in Fig. 12b. Among various oxygen related defects^66^ examined by DFT using hybrid functionals (HSE06), found that B-vacancy with two oxygen atoms (V_B_O_2_) defect structure as shown in Fig. 12c, is the most likely defect for emission in longer wavelength. The emission energy of V_B_O_2_ defect (theoretically calculated) found to be consistent with experimental ZPL energy of near IR emitters fabricated using Ar plasma etching.

Recent studies reveal that boron and nitrogen dangling bonds as shown in Fig. 12d, e as another source of single-photon emission around 2 eV, which is described in ref^131^.

Numerous DFT calculations anticipate that single photon emission in UV region is due to carbon substitutional defects like C_N_ defect (nitrogen atom is replaced with carbon) and C_B_ defect^132^ (boron atom is replaced with carbon). Their quantum emission (theoretical) was observed ~ 4.1 eV, which were consistent with experimental works as described in ref^63^.

By using Finite Difference Time Domain (FDTD) simulations as demonstrated in ref^114^, the enhancement of single photon source emission characteristics due to coupling with gold nanospheres was investigated as shown in Fig. 12f. The simulated spontaneous enhancement rate was consistent with experimental studies. X-polarized emitters which are perpendicular to the gold metal surface exhibits higher enhancement rates than y-polarized emitters.

Strain tunable quantum emission from N_B_V_N_ defect structure as shown in Fig. 12g, was studied using DFT calculations by PBE approximation. Four strain directions were considered in simulations^78^ to create similar effects of strain, induced using polycarbonate (PC) beam in experimental works.

The quantum emission from N_B_V_N_ defect is observed ~ 2.01 eV (black peak), when no strain was applied (zero strain). When tensile strain is applied along **ZZ**_**1**_ direction, the emission peaks were tunable towards lower wavelengths (blue tones) and similarly, when tensile strain applied along AC_2_ direction, the emission peaks were tunable towards higher wavelengths (red tones).

### Applications of quantum emitters

Many applications of quantum emitters have been proposed and demonstrated in literature. Some major applications of quantum emitters are discussed here:

i. Quantum computing,
ii. Quantum cryptography (Quantum communication),
iii. Quantum imaging and metrology and
iv. Other fascinating applications

#### Quantum computing

In classical computing, a conventional bit can be either a 0 or 1. Quantum computer provides a breakthrough revolution, in which a qubit can be both 0 and 1 at the same time on the basis of superposition principle.

In optical quantum computing, the single photons are considered as qubits. Initially, these single photons generated from single photon emitters can be either horizontally polarized (considered as logic 0) or vertically polarized (considered as logic 1). Then processing of qubits like creating superposition state (both logic 0 and 1 at same time) and flipping of qubits (logic 0→logic 1 or logic 1→logic 0) can be created using quantum gates like Hadamard (H) gate and Pauli-X gate etc. respectively. These quantum gates are developed using set of birefringent wave plates and the encrypted qubits finally accumulated using single photon detectors.

**Figure 13:**
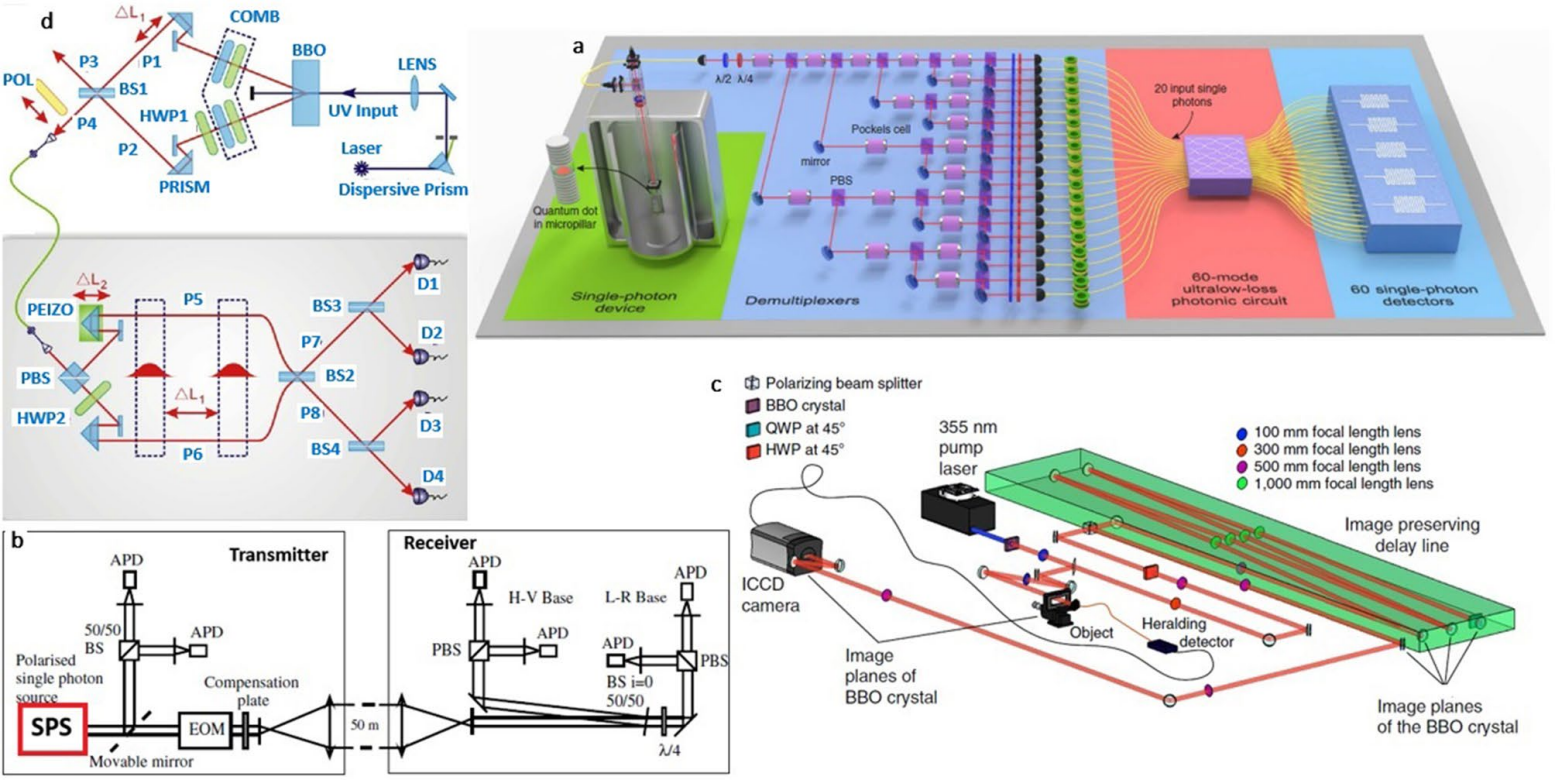
Schematic representation of quantum photonic circuit in a quantum computer^136^, complete quantum communication system for free space propagation^137^, Schematic illustration of quantum imaging and quantum metrology circuits^140,142^. (**a**) The quantum photonic circuit developed for quantum computing application, in which quantum light sources as quantum dots are employed. (**b**) The quantum communication setup in which the single photon source (highlighted in red colour) is present at the transmitter. (**c**, **d**) Circuit schematics for quantum imaging and metrology applications, in which necessary entangled photons are generated by nonlinear BBO (beta barium borate) crystal by spontaneous parametric down conversion technique and separated by a beam splitter, modified Mach–Zehnder interferometer (MZI) (background grey colour) shown in Fig. 13d for photon processing.

The schematic representation of a quantum photonic circuit in a quantum computer, constructed using single photon source, set of quantum gates and polarized beam splitters (PBS) and single photon detectors as shown in Fig. 13a, in this particular example quantum dot (QD) based single photon sources^133–136^ are used for discussion.

#### Quantum cryptography (quantum communication)

Quantum cryptography technique is used to encrypt the key by using quantum effects (polarized photons) for secure communication and the complete quantum communication^137^ setup is shown in Fig. 13b.

The quantum communication setup in which the single photon source (highlighted in red colour) is present at the transmitter, coupled to HBT setup for controlling the quality of source and EOM (electro optical modulator) to make photons polarized. The sender and receiver are separated by a distance of 50 m. The horizontal–vertical (H−V) and circular left–circular right (L−R) polarization basis photons were used for data encryption.

To date, Color centers in diamond^87^ (free space) and Quantum dots^138^ (optical fiber communication to a distance of 120 km) are used as single photon emitters in quantum communication systems.

#### Quantum imaging and metrology

##### Quantum imaging

Quantum imaging is a new sub-field of quantum optics, makes use of entangled photons to image the objects with higher resolution or other imaging criteria beyond the limitations of classical optics.

The simplified schematic of quantum imaging experiment is shown in Fig. 13c in which two entangled photons^139,140^ are generated using BBO (beta barium borate) crystal.

##### Quantum metrology

Similar to quantum imaging application, quantum metrology^141,142^ exploits entangled photons to make high sensitive measurements of assorted signal (weak emissions along with strong unwanted background) and physical parameters in order to avoid statistical errors. The schematic representation of quantum metrology circuit is shown in Fig. 13d.

#### Other fascination applications

##### Other fascinating applications of quantum emitters

Other fascinating quantum technology applications of single photon emitters (detected in 2D materials like hBN, WSe_2_, MoSe_2_ and MoS_2_ etc.) were listed in Table 15.

**Table 15:** Other fascinating quantum applications of single photon emitters.

Applications	2D Materials used	Designing technique	References
Quantum photonic circuits	hBN	Photonic crystal cavities in hBN monolayer	^143^
		Multilayer hBN in confocal microcavity	^144^
	WSe_2_	WSe_2_ monolayer on silicon nitride chip	^145^
		Strained WSe_2_ monolayer coupled to silicon nitride photonic waveguide	^146^
	MoSe_2_	MoSe_2_ layer between two Cr/Au electrodes on Si/SiO_2_	^31^
	MoS_2_	MoS_2_ layer coupled to PVA (Poly vinyl alcohol) film on Si/SiO_2_	^147^
	hBN & Graphene	Graphene-hBN-Graphene heterostructure on Si/SiO_2_	^118^
		Graphene-hBN hyperstructure	^148^
Quantum plasmonic circuits	hBN	hBN monolayer coupled to plasmonic nanocavity arrays	^149^
	WSe_2_	WSe_2_ monolayer coupled to plasmonic nanopillars	^150^
		WSe2 monolayer coupled to metal–insulator-metal waveguide	^151^
Quantum repeaters	hBN	Layered hBN coupled to tapered optical fiber	^152^
Quantum LED	hBN, Graphene & WSe2	Graphene, hBN and WSe2 layers heterostructure on Si/SiO_2_	^58^

The applications of 2D material based single photon emitters in quantum photonic and plasmonic circuits, quantum repeaters and quantum LED and their designing techniques.

Surging the enhancement of emission characteristics in hBN like single photon purity, suppressing off-resonant noise, reduction of excited state lifetime and elimination of photo blinking and bleaching using Purcell effect was perceived by creating an array of plasmonic nanocavities^149^.

Contemporarily, the enhancement without coupling to plasmonic nanoparticles was made feasible using photonic crystal cavities^143^ and microcavities^144^, which enhances the implementation of scaled quantum photonic circuits. As similar to stark tuning of emission in hBN and graphene heterostructure, efficient spontaneous emission and enhancing photon extraction was realized through graphene-hBN hyperstructure^148^.

Coupling of quantum emitters in hBN to tapered optical fibers^152^ escalates the application of on-demand quantum repeaters, but the collection efficiency was observed around 10%.

Deposition of TMDs like WSe2 material on plasmonic nanopillars^150^, originates simultaneous exciton trapping and enhancement in emission characteristics. Recently coupling the WSe2 material to silicon nitride^145,146^ nanochip and waveguide found to increase photon extraction efficiency. Tunable quantum emission due to electric and magnetic fields and control of emitter lifetime in TMDs were also observed^31,147^ as similar to spectral tuning observed in hBN^57,118^. Particularly, the graphene/hBN/TMDs heterostructure facilitated the controlled charge trapping of excitons^58^.

##### Implementation of qubits (single photons) using various 2D materials and heterostructures

**Figure 14:**
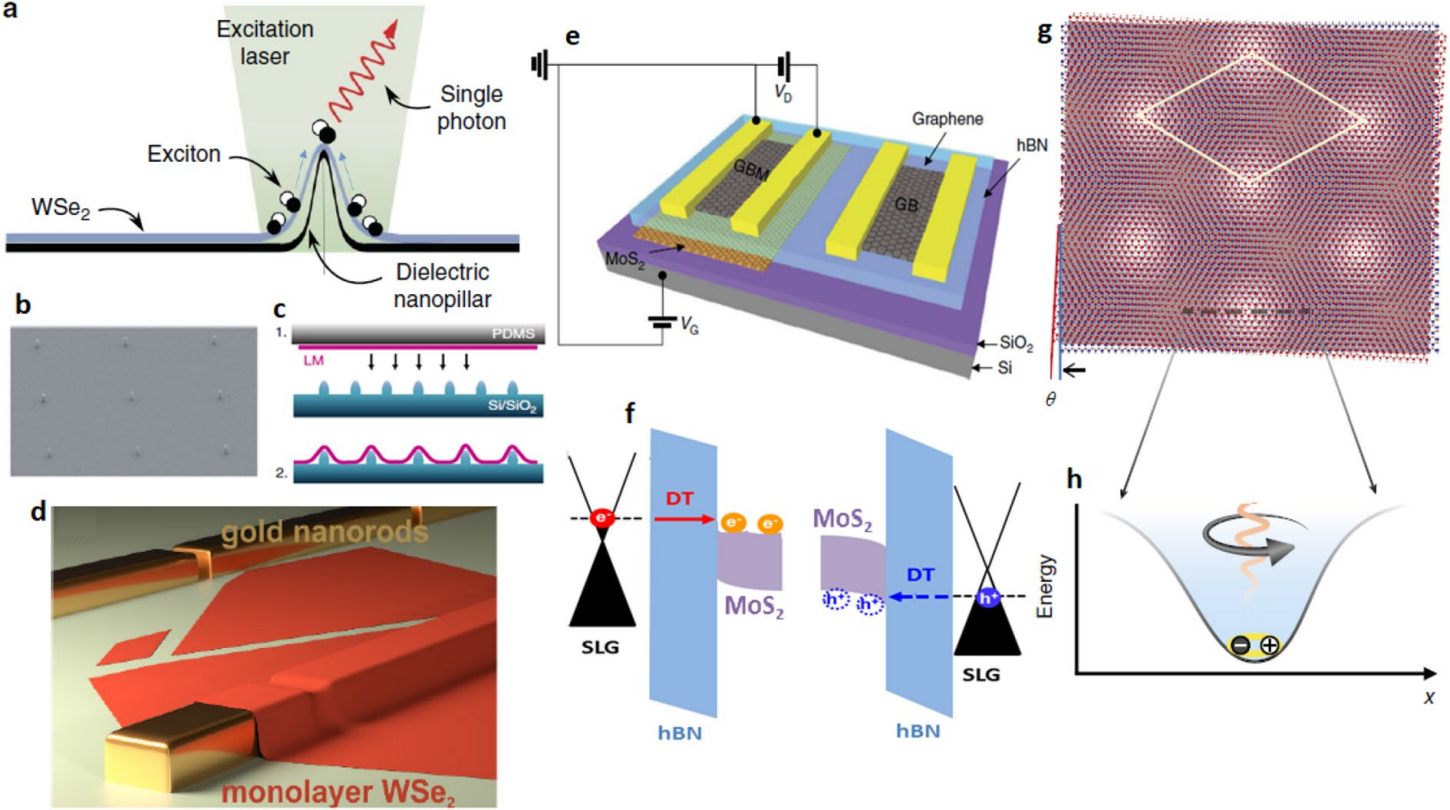
Techniques to implement qubits using other 2D materials and heterostructures^19,29,47,49,58^. (**a**) Intentionally induced strain gradient in WSe_2_ layer to funnel a single exciton using dielectric nanopillar. (**b**, **c**) SEM image of an array of quantum emitters using nanopillars and schematic representation of dry transfer technique of layered material on nanopillars. (**d**) Monolayer WSe_2_ folded around the gapped golden rods which leads to strain inducement and formation of potential wells to effectively trap and localize the excitons. (**e**) graphene/hBN/MoS_2_/hBN/graphene heterostructure for excitonic emission using electrical excitation. (**g**) Illustration of moiré super-lattice due to 2D heterobilayers structure to form electrostatic potential traps. (**h**) Schematic representation of an exciton trapped in a moiré electrostatic potential site.

The implementation of qubits using various 2D materials and heterostructures are listed in Table 16 with relevant discussion and the corresponding figures are shown in Fig. 14.

**Table 16:** Implementation of qubits using 2D materials. Qubits implementation using various 2D materials, bilayers and heterostructures, responsible excitation mechanisms and different emission phenomena and their corresponding detailed explanations.

Emission	Excitation	Materials	Supportive information	References
Localized Excitonic emission associated to point defects and crystal imperfections	Optical	WSe_2_	Point defects and crystal imperfections (local strain) acts as efficient carrier trapping centers, where free excitons are trapped and localized. Intentionally induced strain traps exciton at maximum strain point as shown in Fig. 14a, d. External electric and magnetic fields can control the excitonic emission.	^19–38^ ^40–48^
		MoSe_2_		
		MoS_2_		
		GaSe		
	Electrical	Graphene/hBN/MoX_2_ or WX_2_ /hBN/Graphene (X = S or Se)	TMDs monolayer as exciton recombination layer, hBN as tunnelling barriers and graphene as transparent electrodes as shown in Fig. 14e. Injection of both electrons and holes into recombination layer as explained in Fig. 14f leads to formation of excitons.	^39^ ^58,59^
Localized Excitonic emission associated to moiré pattern	Optical	WSe_2_/WS_2_ heterostructures	Moiré pattern creates an array of nanoscale electrostatic potential that can trap quasiparticles including excitons as shown in Fig. 14g, h. The energy states and the periodicity of the superlattice can be controlled by the twist angle as shown in Fig. 14g. External electric fields can control the excitonic emission.	^49–53^
		MoSe_2_/WS_2_ heterobilayers		
		MoSe_2_/WSe_2_ heterobilayers		
	Electrical	MoS_2_/WSe_2_ heterobilayers		
Spontaneous emission due to intermediate energy state transition	Optical	Layered hBN	hBN as a wide bandgap material, host optically active luminescent point defects that have ground and excited states within the gap. Luminescent point defects are created using ion implantation, laser ablation, electron beam irradiation, Ar plasma etching and various subtler processes and activated with high temperature annealing. Emission from point defects can be controlled using electric and magnetic fields and enhanced using two laser excitations and plasmonic coupling. Tuning of emission can be performed by externally induced strain, electric and magnetic fields.	^56,57^ ^63–66^ ^78^ ^96,98^ ^113^ ^114^

Typical qubits (single photons) can be implemented by 2D materials using various phenomena like spontaneous emission associated to intermediate energy state transition in hBN, spontaneous emission associated to localized excitons trapped and funneled due to point defects and crystal imperfections in TMDs.

Electrostatic potential traps due to moiré patterns and intentionally induced strain gradients can also effectively tap excitons for qubits implementation.

Especially, in case of WSe_2_ material the crystal imperfections due to intentionally induced strain gradients by transferring on dielectric nanopillars and folding the material using gapped golden rods as shown in Fig. 14a, d, which leads to funnel and trap excitons. Optically exciting these trapped excitons leads to single photon emission. In similar fashion, an array of quantum emitters can be created by trapping the excitons using set of dielectric nanopillars as shown in Fig. 14b, the corresponding dry transfer technique of layered material on array of nanopillars in shown in Fig. 14c.

Moiré super-lattice of 2D heterobilayers structure as shown in Fig. 14g with twist angle θ leads to formation of electrostatic potential traps for effective trapping of exciton as shown in Fig. 14h.

Qubits can also be implemented by electrical excitation of excitons using 2D heterostructures like graphene/hBN/MoS_2_/hBN/graphene heterostructure as shown in Fig. 14e. The MoS_2_ acts as recombination layer, hBN as tunneling barrier and graphene as transparent electrodes. To verify the effect of charge trapping complete structure is divided into two: one with MoS_2_ layer (GBM) and without MoS_2_ layer (GB).

Energy band structure of graphene/hBN/MoS_2_/hBN/graphene heterostructure is shown in Fig. 14f. By increase in applied voltage, quasi fermi level of graphene (left contact) reaches minimum of conduction band of MoS_2_, electrons tunnel through the hBN barrier into MoS_2_ layer and quasi fermi level of graphene (right contact) reaches maximum of valence band of MoS_2_, holes tunnel through the hBN barrier into MoS_2_ layer. Injection of electrons and holes in MoS_2_ layer (recombination layer) leads to formation of exciton recombination followed by single photon emission.

Electrical manipulation of excitonic emission to generate entangled photons^59–61^ using 2D heterostructures, as similar to shown in Fig. 14e, is also realized which facilitates the application of quantum imaging and metrology.

#### Practical challenges of implementing quantum emitters and their applications

Few major challenges are noticed in fabricating hBN single photon emitters and developing towards quantum applications. The challenges associated with emitters (luminescent point defects) hosts in hBN were listed in Table 17.

**Table 17:**
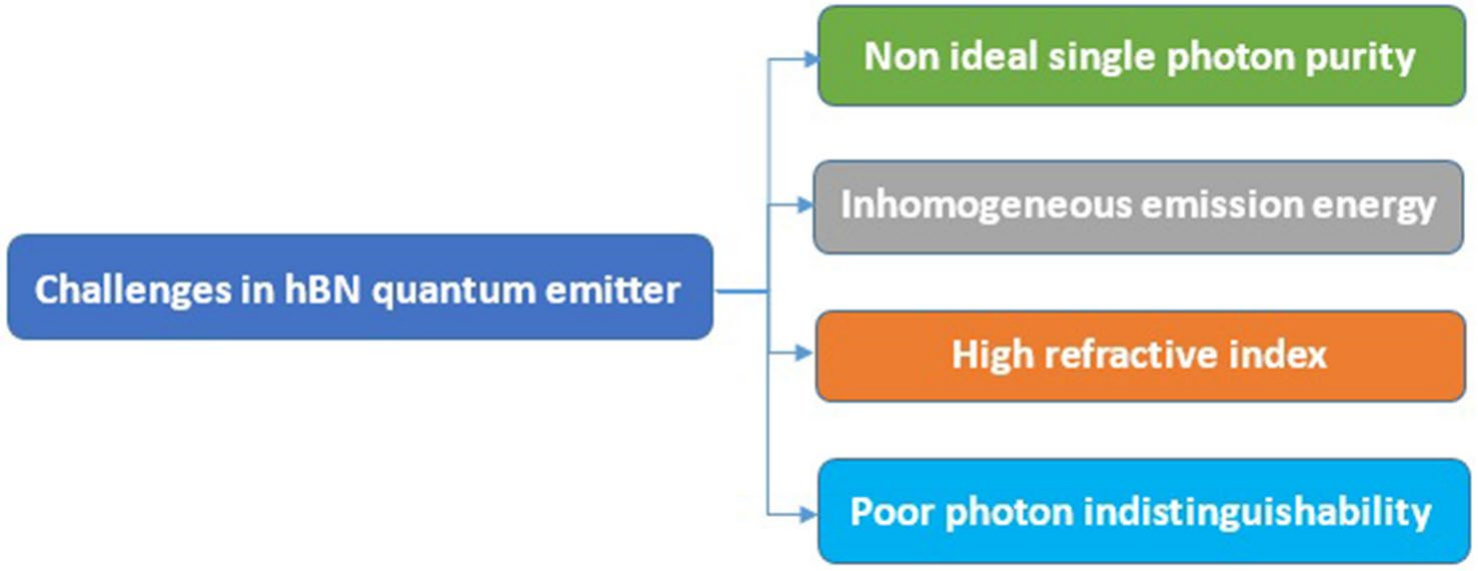
The challenges in fabricating ideal hBN single photon emitters.

One of the major challenge to be resolved is moderate single photon purity (non-ideal) due to residual background emission. In most of the experiments it was noted that emission intensity of emitters spans over a large spectral band (inhomogeneous spectral distribution) which is a central problem for developing identical single photon sources.

High refractive index of the material which makes difficult to confine the light within the hBN structure is another important problem to be addressed and finally, indistinguishability of photons from the emitter is very poor.

The challenges associated with optical quantum applications were listed in Table 18. In quantum computing, the loss of quantum coherence due to temperature fluctuations is the one major challenge to be overcome for practical implementation and another problem is influence of other electromagnetic waves and undesirable interactions with outer environment leads to collapse of quantum properties of computer.

**Table 18:**
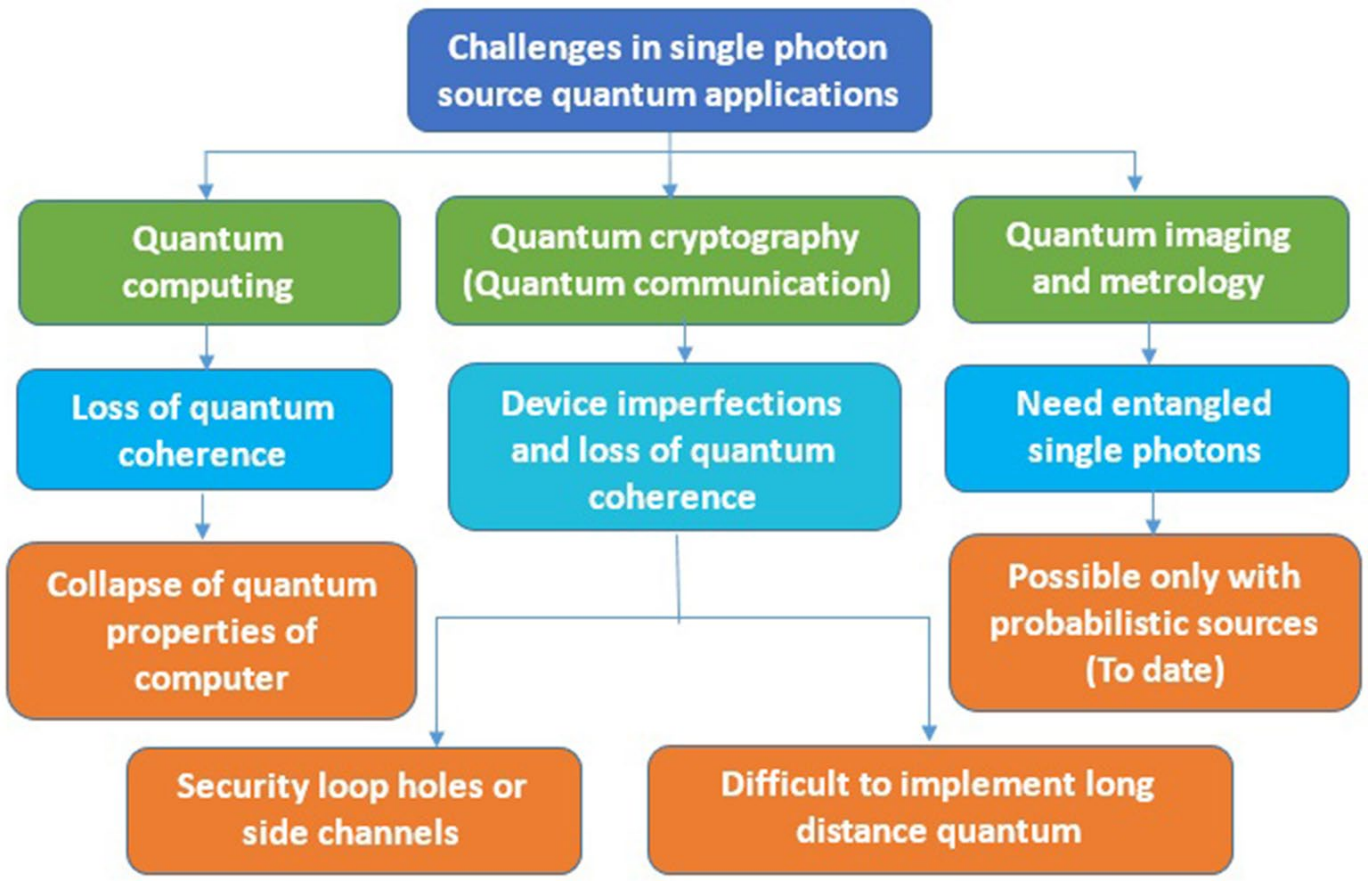
The challenges in implementing quantum applications using single photon sources.

In case of quantum communication, Security loopholes or side channels leads to in-secure quantum communication. Currently, probabilistic single photon sources are used for entangle photon generation which are not reliable for on-demand applications like quantum imaging and metrology.

The complete summary of hBN materials used and their synthesis, stable emitter formation techniques, defects consistent with experimental studies, important photophysical characteristics of emitters and their emission enhancement techniques and major applications of quantum emitters were discussed in Table 19.

**Table 19:**
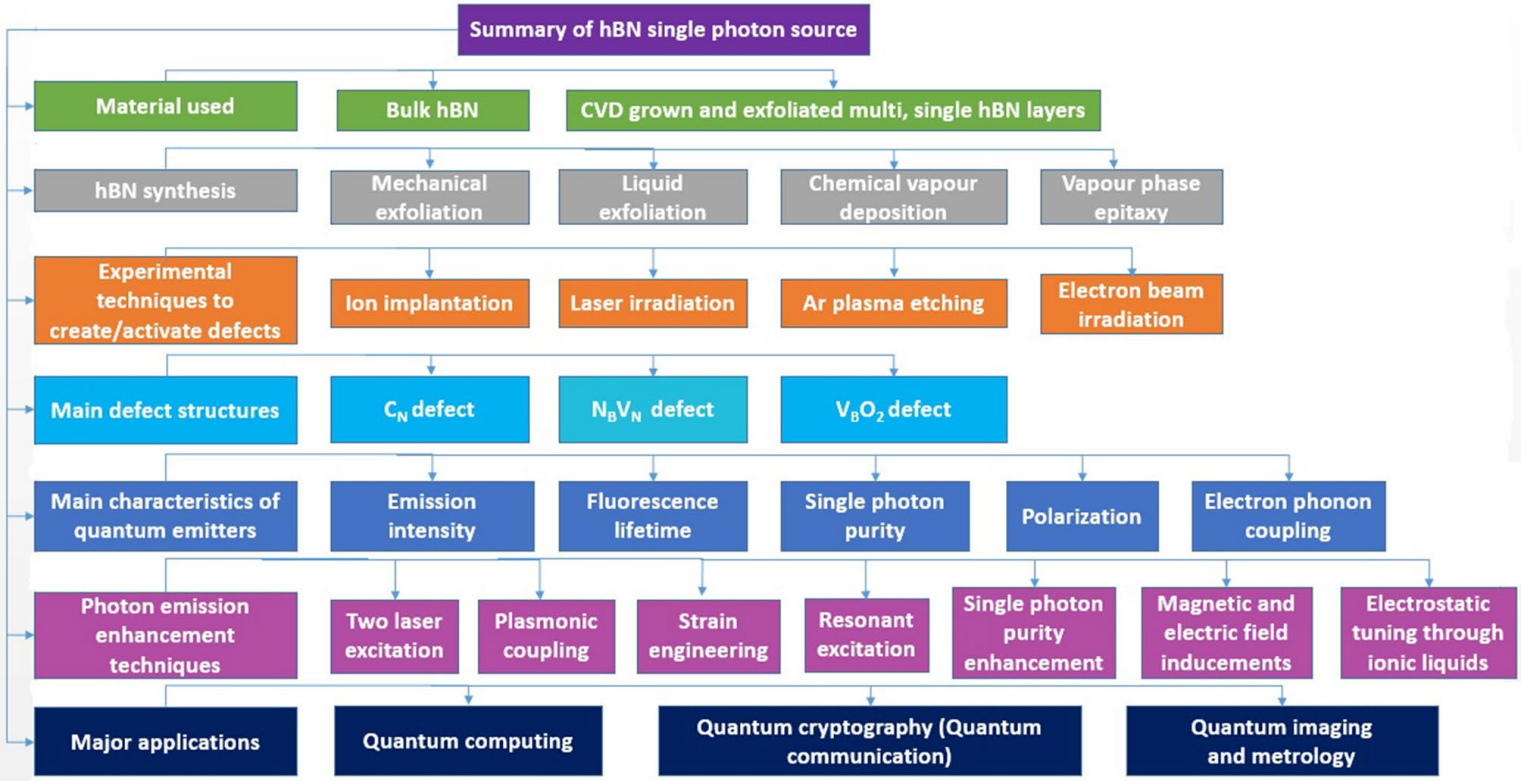
Overall summary of hBN based single photon quantum emitters.

The complete summary of hBN single photon sources.

## Conclusion and outlook

Single photon emission is observed from single atom in a cavity to recent 2D materials, however, each of these sources have their own constraints. However, the state-of-the-art quantum emitters in 2D materials found to be an effective alternate to traditional single photon emitters in three dimensional materials due to the disadvantage of photon extraction efficiency and external circuit coupling.

In the quest of A1 supreme quantum emitters, strong excitonic single photon emission is observed in monolayer TMDs^19–46^ and in their Moiré patterns^49–53^ at cryogenic temperatures. Despite, inducing a stronger strain in TMDs is found to improve their operating temperatures^22^ and quantum yield^47,48^. It’s an auspicious that emitters observed in multilayer hBN are found to be stable at a wide range of operating temperatures, various harsh environments and found to be localized at flake edges in mechanically exfoliated flakes which enhances the device fabrication and coupling to plasmonic cavity for higher integration quantum photonic circuits.

This solid quantum emitters in hBN are found to be generated by Ion implantation, laser ablation, Ar plasma etching followed by subsequent annealing and electron beam irradiation. But, the nature of emitters formed is still under debate. The cutting-edge research is fabricating stable quantum emitters in higher order (⁓ 100–200 SPEs per 10 × 10 μm^2^) in large few-layer hBN fabricated with LPCVD technique, strongly publicized that more than 85% of the emitters have a ZPL at (580 ± 10) nm, which is the confined uneven spectral distribution reported to date^97^.

To enhance the performance of quantum photonic devices and converge to on-demand applications, fabricating the quantum emitters which covers the wide range of emission on a single platform is really worthy of attention. Significantly, hBN exhibits broad emission range from UV to near IR region and the first signature of Rabi oscillations and resonant fluorescence emission was observed from a resonantly drive hBN quantum emitter^153^. Contemporarily, an attractive phenomenon of second harmonic generation (SHG)^119,120^ was discovered in hBN multilayer coupled to circular Bragg grating (CBG) photonic microstructures which, encourages the delightful applications.

Although the emission characteristic measurements in hBN were not up to the mark, quantification enhancement techniques like Two laser excitation technique, plasmonic coupling using gold nanospheres, external strain engineering, electrostatic tuning through ionic liquid devices^97^ and very recently electric and magnetic field inducements^57^ etc., techniques found to enhance and tune the quantum emitters emission properties and control the inhomogeneous spectral distribution. To date a record tuning of quantum emission around 65 meV^124^ and higher rate of emission enhancement (around 6 to 15 fold) owing to coupling with photonic crystal cavities from silicon nitride (Si_3_N_4_)^121^ and Al nano-antenna^122^ was perceived. However the quantum emitters in hBN experience spectral diffusion at cryogenic temperatures, Resonant and antistokes^154^ excitation technique found to overcome the complication due to spectral diffusion.

Emitters in hBN are mostly stable under illumination at 532 nm. On the other facet, emitters exhibits fluorescence instability and spectral diffusion at blue laser excitation^155^. However employing different substrates like Al_2_O_3_^156^ or InGaP^157^ found to reduce the random spectral fluctuations, layered hBN mounts a problem of invisibility on opaque substrates under white light illumination. Recent explorations make a Serendip that hBN was found to enhance the contrast on transparent substrates^123^, which makes experimental works feasible on hBN.

Efficient photon extraction and coupling to external photonic circuits and devices is at most important for practical implementation for quantum applications. Out of sort, only 10% efficiency in photon extraction using an optical fiber and coupling to an optical waveguide is achieved^146^ at room temperature. A waveguide of proper design is needed to improve the coupling of quantum emitters and filter the propagating laser light, to enhance the feasible implementation of quantum repeaters.

As a quantum breakthrough, tunable excitonic emission in hybrid structures initiate the development of quantum memories^58^. Entanglement of photons for Quantum imaging and metrology was made possible by a back-gated WSe_2_ monolayer, hBN and graphene heterostructure^59–61^, overcome the drawback of generating entangled photons at random process in nonlinear BBO crystal.

Several computational evidences disclose that point defects responsible for single photon emission. Investigation of 35 different likely possible point defect structures by density-functional theory (DFT) provides a strong evidence that N_B_V_N_ defect structure is more consistent with the emitter responsible for visible region emission, V_B_O_2_ defect for near IR region and C_N_ defect for UV emission. Recent theoretical studies reveal boron dangling bonds^125^ are also the likely source of emission around 2 eV and C_B_ defect^126^ as another candidate responsible for UV emission.
