# Publisher Correction: Optical quantum technologies with hexagonal boron nitride single photon sources

**DOI:** 10.1038/s41598-021-95271-5

**Published:** 2021-08-13

**Authors:** Akbar Basha Dhu‑al‑jalali‑wal‑ikram Shaik, Penchalaiah Palla

**Affiliations:** Center for Nanotechnology Research & Department of Micro and Nanoelectronics, School of Electronics Engineering, Vellore Institute of Technology (VIT), Vellore, Tamil Nadu 632014 India

Correction to: *Scientific Reports* 10.1038/s41598-021-90804-4, published online 10 June 2021

The original version of this Article contained errors.

Affiliation 1 was incorrectly given as ‘Center for Nanotechnology Research/Department of Micro and Nanoelectronics, School of Electronics Engineering, Vellore Institute of Technology (VIT), Vellore, Tamil Nadu, 632014, India’. The correct affiliation is listed below:

Center for Nanotechnology Research & Department of Micro and Nanoelectronics, School of Electronics Engineering, Vellore Institute of Technology (VIT), Vellore, Tamil Nadu, 632014, India.

Additionally, Figure 3 and Figure 4 contained incorrect reference numbers. The original Figure 3 and Figure 4 appear below with their accompanying legends.

**Figure 3:**
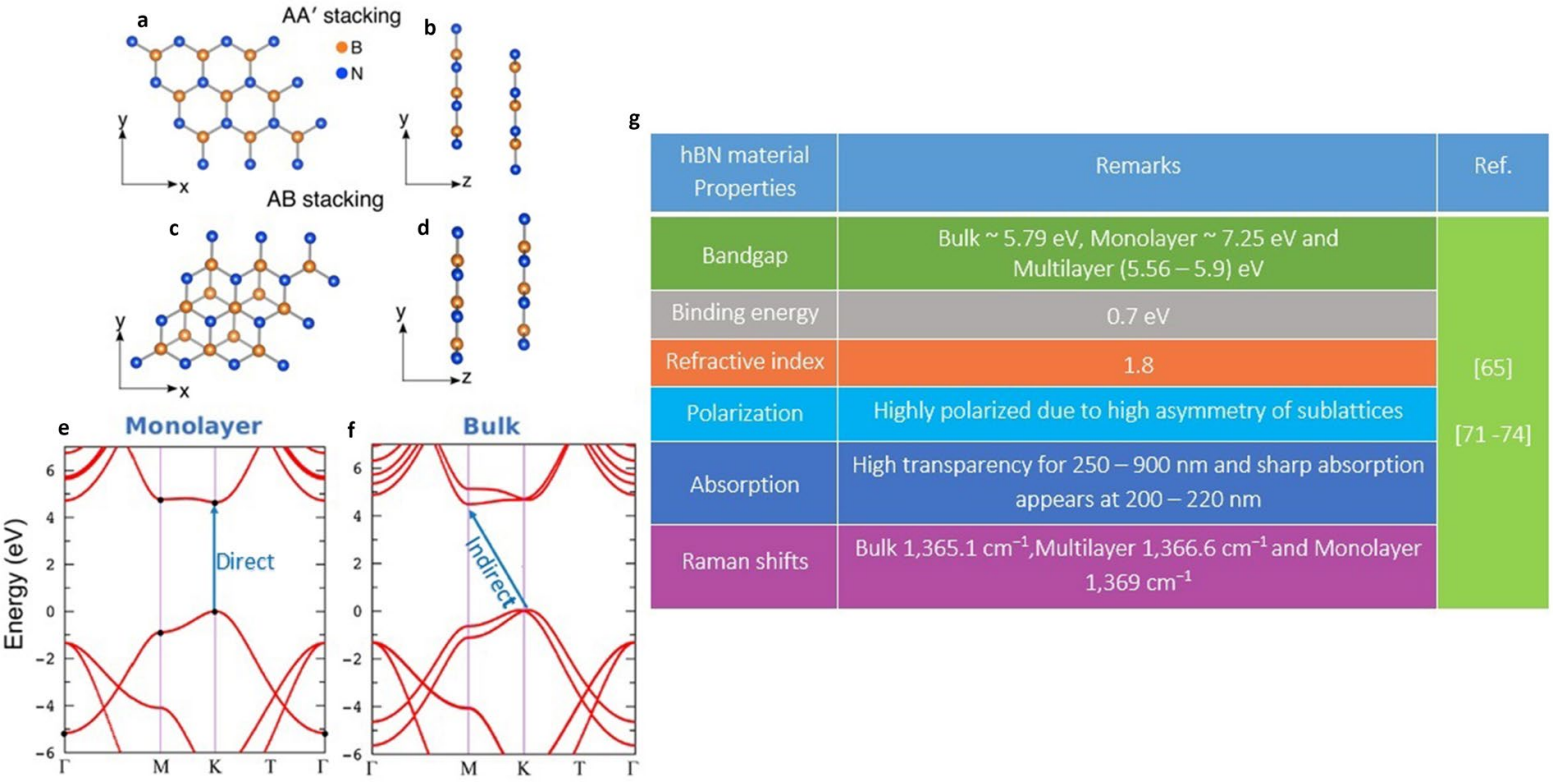
Schematic representation of hBN stacking^74^, electronic band structure of monolayer and Bulk hBN^75^ and electrical/optical/crystal properties of hBN material. (**a**,**b**) Top view and side view of AA’ stacking. (**c**,**d**) Top view and side view of AB stacking. (**e**,**f**) Electronic band structure of monolayer and bulk hBN with direct and indirect bandgaps respectively. (**g**) General properties of hBN material and similar Raman shifts (around values) can be observed for high quality crystals.

**Figure 4:**
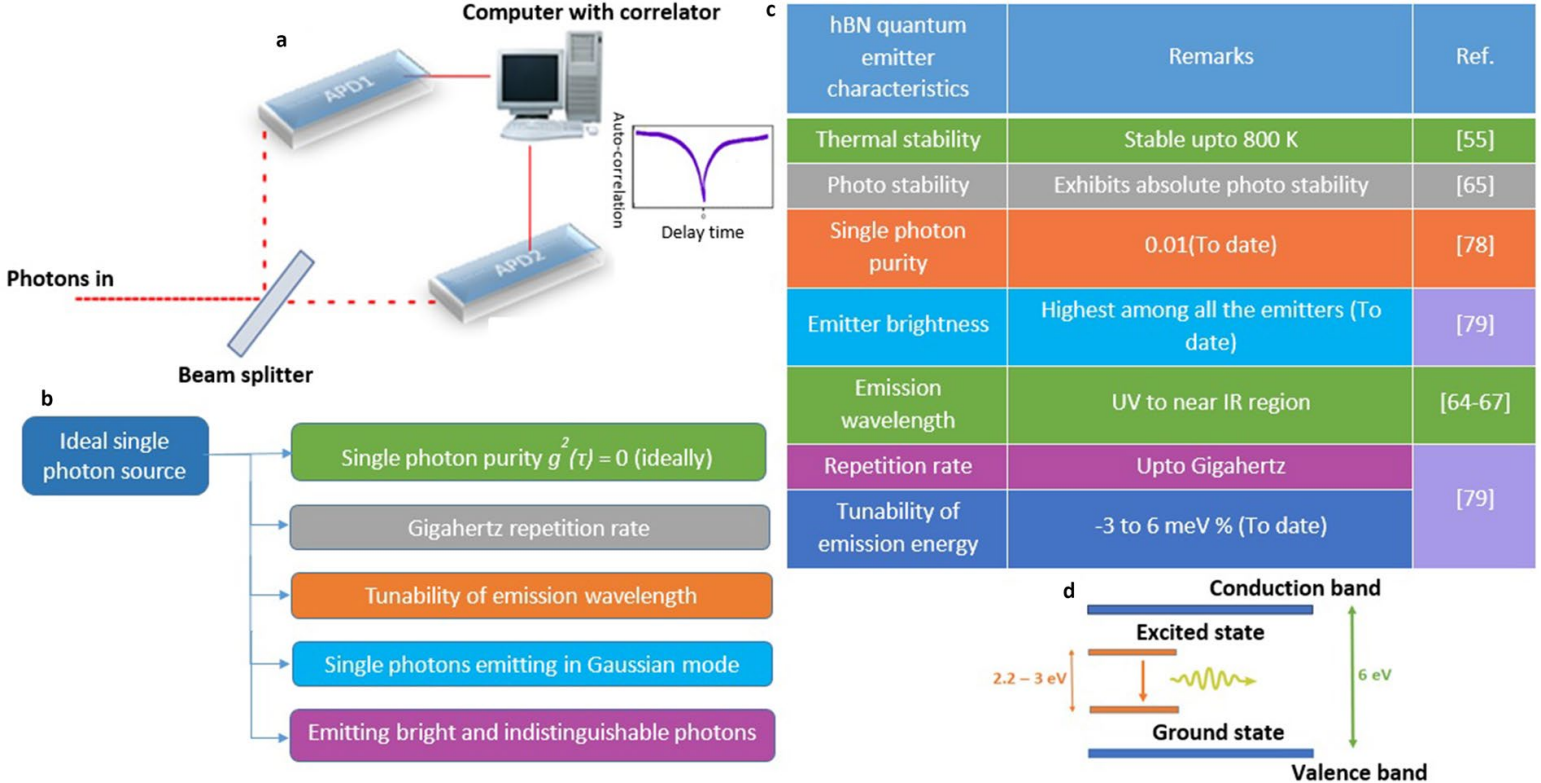
Schematic of HBT interferometer, important features of an ideal single photon source and experimentally observed quantum emitter characteristics, pictographic representation of atomic behaviour of defects within the host bandgap **(a)** Schematic representation of HBT interferometer working mechanism and resultant second order autocorrelation curve representing characteristics of a single photon emitter. **(b)** Important features of an ideal single photon source. **(c)** Experimentally observed some of the quantum emitter characteristics hosts in hBN, in which characteristic stability upto 800 K and single photon purity 0.01 makes a highest record among all the 2D materials (to date). **(d)** The energy band diagram of an hBN host with ~ 6 eV bandgap. A luminescent point defect in hBN (with energy range ~ 2.2–3 eV) exhibits an artificial atom kind of behaviour with ground and excited states.

Also, Table 13 was mistakenly duplicated in the place of Figure 13, causing Figure 13 to be omitted from the Article.

Furthermore, Table 13 and Table 16 contained errors in the merging of the columns, as well as in the references. The original Table 13 and Table 16 appear below with their accompanying legends.

**Table 13:**
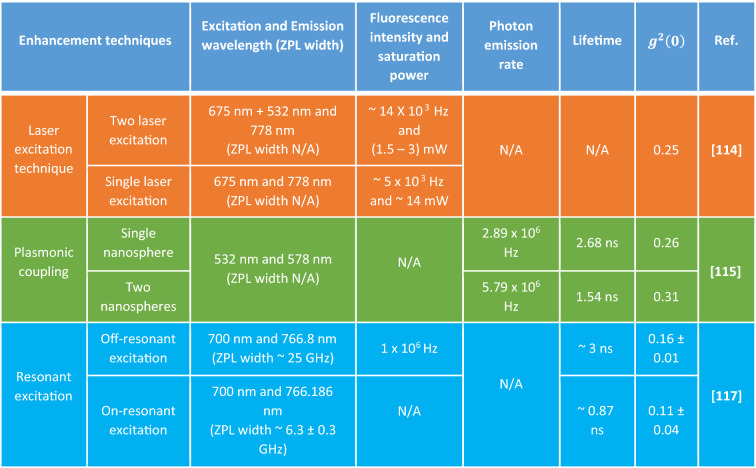
Intensified photophysical characteristics of emitters due to three different enhancement techniques. Enhancement in fluorescence intensity and reduction of excitation saturation power for two laser excitation technique has shown in first row; increase in photon emission rate and controlled fluorescence lifetime due to two nanospheres plasmonic coupling. Modulated ZPL (wavelength and width); enhanced single photon purity and controlled fluorescence lifetime due to on-resonant excitation is shown in third row.

**Table 16:**
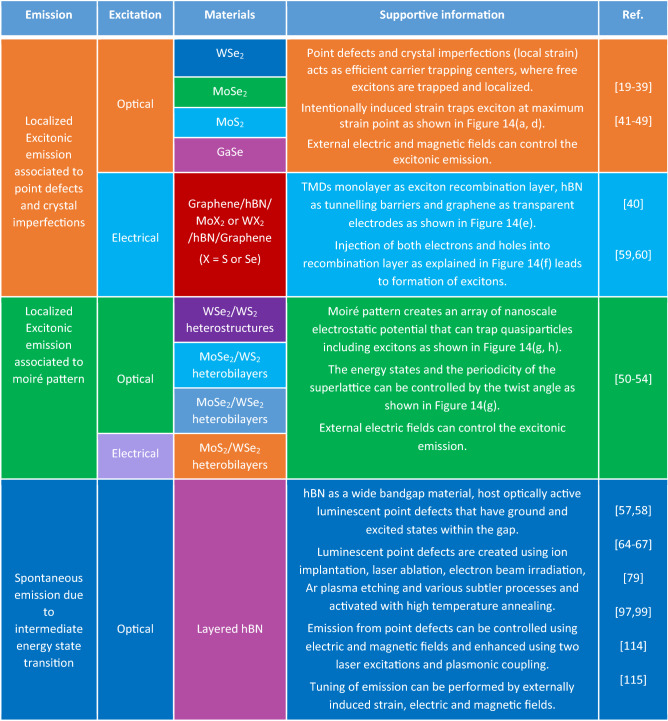
Implementation of qubits using 2D materials. Qubits implementation using various 2D materials, bilayers and heterostructures, responsible excitation mechanisms and different emission phenomena and their corresponding detailed explanations.

Finally, Tables 2, 10, 17, 18 and 19 were updated to give layout uniformity.

The original Article has been corrected.

